# Molecular and functional imaging in cancer-targeted therapy: current applications and future directions

**DOI:** 10.1038/s41392-023-01366-y

**Published:** 2023-02-27

**Authors:** Jing-Wen Bai, Si-Qi Qiu, Guo-Jun Zhang

**Affiliations:** 1 Fujian Key Laboratory of Precision Diagnosis and Treatment in Breast Cancer, Xiang’an Hospital of Xiamen University, School of Medicine, Xiamen University, 361100 Xiamen, China; 2 Xiamen Key Laboratory of Endocrine-Related Cancer Precision Medicine, Xiang’an Hospital of Xiamen University, School of Medicine, Xiamen University, 361100 Xiamen, China; 3 Xiamen Research Center of Clinical Medicine in Breast and Thyroid Cancers, Xiang’an Hospital of Xiamen University, School of Medicine, Xiamen University, 361100 Xiamen, China; 4 Department of Breast-Thyroid-Surgery and Cancer Center, Xiang’an Hospital of Xiamen University, School of Medicine, Xiamen University, 361100 Xiamen, China; 5 Department of Medical Oncology, Xiang’an Hospital of Xiamen University, School of Medicine, Xiamen University, 361100 Xiamen, China; 6 Cancer Research Center of Xiamen University, School of Medicine, Xiamen University, 361100 Xiamen, China; 7 Diagnosis and Treatment Center of Breast Diseases, Clinical Research Center, Shantou Central Hospital, 515041 Shantou, China; 8 Guangdong Provincial Key Laboratory for Breast Cancer Diagnosis and Treatment, Shantou University Medical College, 515041 Shantou, China

**Keywords:** Cancer imaging, Cancer therapy

## Abstract

Targeted anticancer drugs block cancer cell growth by interfering with specific signaling pathways vital to carcinogenesis and tumor growth rather than harming all rapidly dividing cells as in cytotoxic chemotherapy. The Response Evaluation Criteria in Solid Tumor (RECIST) system has been used to assess tumor response to therapy via changes in the size of target lesions as measured by calipers, conventional anatomically based imaging modalities such as computed tomography (CT), and magnetic resonance imaging (MRI), and other imaging methods. However, RECIST is sometimes inaccurate in assessing the efficacy of targeted therapy drugs because of the poor correlation between tumor size and treatment-induced tumor necrosis or shrinkage. This approach might also result in delayed identification of response when the therapy does confer a reduction in tumor size. Innovative molecular imaging techniques have rapidly gained importance in the dawning era of targeted therapy as they can visualize, characterize, and quantify biological processes at the cellular, subcellular, or even molecular level rather than at the anatomical level. This review summarizes different targeted cell signaling pathways, various molecular imaging techniques, and developed probes. Moreover, the application of molecular imaging for evaluating treatment response and related clinical outcome is also systematically outlined. In the future, more attention should be paid to promoting the clinical translation of molecular imaging in evaluating the sensitivity to targeted therapy with biocompatible probes. In particular, multimodal imaging technologies incorporating advanced artificial intelligence should be developed to comprehensively and accurately assess cancer-targeted therapy, in addition to RECIST-based methods.

## Introduction

Cancer is the leading cause of mortality globally. It was estimated that approximately 2,370,000 and 4,820,000 new cancer cases and 640,000 and 3,210,000 cancer deaths would occur in the United States and China in 2022, respectively.^1^ Surgery and radiotherapy (RT) are two primary treatment cornerstones of locoregional and nonmetastatic cancers, while chemotherapeutics can be used in all cancer stages. Current chemotherapeutics are often limited by undesirable side effects due to an inability to distinguish between tumorous and normal tissues. To mitigate these side effects, it is possible to develop targeted therapy using monoclonal antibodies or small-molecule inhibitors directed against specific signal transduction pathways for angiogenesis, proliferation, survival, and invasiveness, which are often dysregulated in tumor cells.^2^ The development of targeted therapies is thus a valuable advance for cancer treatment.

To evaluate the efficacy of anticancer treatment, both the World Health Organization (WHO) response criteria^3^ and Response Evaluation Criteria in Solid Tumors (RECIST)^4^ utilize changes in tumor size as determined using imaging techniques such as CT, MRI, and positron emission tomography (PET). RECIST 1.1 is currently the gold standard for assessing treatment response in solid tumors in a clinical context, but it is insufficient for some targeted medications such as antiangiogenic agents and immunotherapy.^5^ For immunotherapy, a 5% rate of pseudo-progression, involving the tumor regressing after initial disease progression or the appearance of new lesions, was reported in non-small-cell lung cancer (NSCLC) patients treated with nivolumab.^6^ According to reports, this phenomenon was caused by an insufficient immune response or edema and necrosis related to immune-cell infiltration into tumor tissue.^7^ RECIST 1.1 relies solely on tumor size and does not consider changes in cellular events such as apoptosis, inhibition of proliferation, cell cycle arrest, tumor metabolism within the tumor microenvironment, and the density and number of intra-tumoral vessels. Thus, it remains unclear how best to evaluate the efficacy of targeted therapies and optimize the therapeutic strategy.

New functional and molecular imaging biomarkers are being developed to better evaluate targeted therapy’s effects. Molecular imaging combines biomedical imaging and molecular biology to visualize and quantify the spatiotemporal distribution of biological processes within living organisms in a noninvasive way for biochemical, biologic, diagnostic, and therapeutic applications.^8,9^ Representative examples of molecular imaging techniques include radionuclide imaging (PET), single-photon emission computed tomography (SPECT), molecular magnetic resonance imaging (mMRI), magnetic resonance spectroscopy (MRS), optical imaging (optical bioluminescence, optical fluorescence), photoacoustic imaging, and multimodal imaging. Some modalities, such as radionuclide and optical imaging, require the injection of molecular probes to acquire the imaging signal. In contrast, mMRI and photoacoustic imaging can track drug effectiveness through endogenous molecules or exogenous molecular probes.

This review focuses on how to use novel imaging modalities to visualize the response to cancer-targeted therapies. Signaling pathway-based targeted therapies are concisely summarized. In addition, functional and molecular imaging modalities are discussed in detail about basic principles, imaging probes, and their application in targeted therapies against different molecular pathways. Lastly, future directions for molecular imaging in targeted therapies are prospectively reviewed.

## Targeted therapies and anticancer drugs

Dysregulation of oncogenic signaling pathways plays a key role in the occurrence and progression of cancer. Substantial efforts have been made in treating cancer through “targeted” therapies that specifically disrupt pro-oncogenic signaling pathways. Specifically, there are two types of targeted therapy: small-molecule (enzyme)-based therapies,^10^ such as with tyrosine kinase inhibitors (TKIs) like sunitinib, and antibody-based targeted therapies,^11^ such as with vascular endothelial growth factor (VEGF)-targeted antibodies including bevacizumab. Cancer immunotherapies, such as immune checkpoint inhibitors (ICIs), targeting the interaction between cancer and immune cells, broaden the scope of targetable tumors.^12^ In this review, we introduce targeted therapies according to each signaling pathway.

### VEGF/VEGFR signaling pathway

Angiogenesis is a crucial step in the successful growth, invasion, and metastasis of tumors, without which tumors could not grow beyond 1–2 mm in diameter.^13^ Multiple growth factors and their receptors are dysregulated in the complex process of tumor angiogenesis. The VEGF/VEGF receptor (VEGFR) signaling pathway is the pivotal inducer of angiogenesis, so antiangiogenic approaches have primarily focused on inhibiting this pathway.

A large number of drugs have been developed for targeting the VEGF/VEGFR signaling pathway^14^: (1) ligand binding agents that block the binding of VEGF ligands to receptors, such as bevacizumab,^15^ which binds to VEGF alone, and aflibercept,^16^ which binds to VEGF and placental growth factor (PlGF); (2) antibodies that block signaling through VEGFR, such as ramucirumab^17^ that targets VEGFR2; and (3) small‐molecule TKIs that block the kinase activity of VEGFR,^18^ such as lenvatinib, sorafenib, sunitinib, pazopanib, and regorafenib. TKIs can often inhibit the activity of other receptor tyrosine kinases, such as platelet-derived growth factor receptors (PDGFRs), fibroblast growth factor receptors (FGFRs), and epidermal growth factor receptors (EGFRs).

Besides, integrin α_V_β3 is highly expressed in tumors and neovascular endothelial cells^19^ and is recognized as an ideal marker for distinguishing between cancerous and normal states. RGD (Arg-Gly-Asp) is a polypeptide including cyclic and linear categories screened by phage peptide display technology, which can specifically bind to the extracellular region of the α chain of α_V_β3. Recently, many studies have reported that RGD peptides can carry drugs to tumor sites precisely and described that radionuclide-labeled RGD peptides have many uses for tumor imaging and therapy.^20^

### EGFR signaling pathway

The epidermal growth factor receptor (EGFR) is a member of the ERBB receptor tyrosine kinase family consisting of EGFR/human epidermal growth factor receptor 1 (HER1), HER2, HER3, and HER4.^21^ EGF ligand binding to EGFR results in EGFR dimerization and activation of intracellular kinase activity. Autophosphorylation of tyrosine residues of EGFR activates two main downstream signaling pathways,^22^ PI3K/AKT and RAS/RAF/MEK/ERK, which regulates cell proliferation, differentiation, and survival.^23^

Inhibitors of the EGFR signaling pathway include small-molecule TKIs and anti-EGFR monoclonal antibodies (MoAbs). TKIs act on the ATP binding pocket of EGFR, inhibit EGFR autophosphorylation and antagonize tyrosine kinase signal transduction.^24^ First-generation (erlotinib, gefitinib, and icotinib) and second-generation (afatinib and dacomitinib) EGFR TKIs have been approved for the treatment of advanced NSCLC patients harboring EGFR-activating mutations.^25^ Unfortunately, resistance is inevitably acquired in most patients, at a median of 10–14 months after treatment.^26^ The most common reason for acquired resistance is the T790M mutation in exon 20 of EGFR. Third-generation mutation-selective EGFR TKIs,^27^ such as rociletinib,^28^ osimertinib,^29^ and almonertinib,^30^ have been developed to overcome this resistance mutation.

The related anti-EGFR antibodies target the EGFR extracellular domain and competitively bind to receptors, which impedes dimer formation, thereby inhibiting intracellular signal transduction.^31^ The antibodies against EGFR include nimotuzumab,^32^ panitumumab,^33^ matuzumab,^34^ and cetuximab.^35^ Antibodies are specific to EGFR, while TKIs can cross-link with other EGFR tyrosine kinases (HER2 and HER4).

### HER2 signaling pathway

Unlike other members of the EGFR family, HER2 has no identified ligand. The HER2 signaling pathway is mediated by its heterodimeric form, created by HER2’s binding to other members of the EGFR family. This heterodimer can transactivate HER2 tyrosine kinase activity, further activating its downstream signaling pathways like PI3K/Akt/mTOR, MAPK, phospholipase C, and protein kinase C.^36^

Numerous HER2 inhibitors had been developed in the last few decades, primarily consisting of monoclonal antibodies (MoAbs), small-molecule TKIs, and antibody–drug conjugates (ADCs).^37^ Trastuzumab, pertuzumab, ertumaxomab, and margetuximab are common monoclonal antibodies targeting HER2. Various HER1/HER2 TKIs, pan-HER TKIs, and dual HER2/VEGF TKIs are in different stages of clinical trials or clinical practice.^38^ Currently, the most commonly used TKI drugs include lapatinib, neratinib, pyrotinib, and tucatinib. HER2 ADCs direct drug delivery to HER2-expressing cancer cells while limiting exposure to normal tissue.^39^ The currently available ADCs for HER2-positive cancer include trastuzumab emtansine (T-DM1) and trastuzumab deruxtecan (T-DXd).

In recent years, several novel emerging groups of anti-HER2 agents, including antibody-based fragments (Fabs), diabodies, minibodies, nanobodies (Nbs), and affibodies, have been explored for HER2-positive breast cancer imaging and targeted radionuclide therapy.^40^

### CDK4/6 signaling pathway

The binding of cyclin D1 to CDKs (CDK4 and CDK6) drives G_1_ to S phase transition and disease progression in tumors.^41^ CDK4/6 inhibitors prevent RB1 phosphorylation and E2F transcription and thereby induce G_1_ cell cycle arrest and block cancer progression. There are three CDK4/6 inhibitors, palbociclib, ribociclib, and abemaciclib, that are approved for treating advanced HR-positive (HR+), HER2-negative (HER2−) breast cancer, either combined with an aromatase inhibitor (AI) as a first-line treatment option or combined with fulvestrant as a second-line treatment option. Among these, abemaciclib is the first US Food and Drug Administration (FDA)-approved CDK4/6 inhibitor for adjuvant therapy in HR^+^ HER2^−^ early-stage breast cancer.^42^

### PI3K/Akt/mTOR pathway

The phosphatidylinositol-3 kinase (PI3K)/Akt/mammalian target of rapamycin (mTOR) signaling pathway is frequently activated in response to various extracellular stimuli, such as growth factors, hormones, and cytokines.^43–45^ PI3K is mainly triggered by the two largest groups of membrane receptors: receptor tyrosine kinases (RTKs) and G-protein-coupled receptors (GPCRs).^46^ Activated PI3K phosphorylates phosphatidylinositol 4,5-bisphosphate (PIP2), which is converted to phosphatidylinositol 3,4,5-triphosphate (PIP3). PIP3 binds to Akt and promotes Akt phosphorylation.^47^ Then, phosphorylated Akt triggers the downstream effector mTOR and results in gene transcription to facilitate cell growth and metabolism, motility, and angiogenesis and suppress apoptosis. Besides, activated mTORC2 (mTOR complex 2) can also promote the hyperactivation of Akt by phosphorylating Akt.^48^ Two tumor suppressors [phosphatase and tensin homolog (PTEN)^49^ and inositol polyphosphate 4-phosphatase type II (INPP4B)]^50^ prevent the activation of downstream of PI3K by dephosphorylating PIP3 and PIP2.^51,52^

The PI3K/AKT/mTOR pathway is often genetically altered in different human cancers.^53,54^ Although many developed small-molecule inhibitors target this pathway, only a few have been approved by the FDA for therapeutic use.

Some isoform-specific inhibitors of PI3K have been approved for treating lymphoma/leukemia and breast cancer,^55^ including a pan-PI3K inhibitor (copanlisib/BAY 80-6946/Aliqopa),^56^ a dual PI3Kγ/δ inhibitor (duvelisib/IPI-145/Copiktra), an α-selective PI3K inhibitor (alpelisib/NVP-BYL719/Piqray),^57^ and a δ-selective PI3K inhibitor (umbralisib/TGR-1202, idelalisib/CAL-101/GS-1101/Zydelig).^58^

As the critical effector of the PI3K/Akt/mTOR pathway, genetic alterations of Akt or its abnormal expression initiate tumor development and lead to resistance to chemotherapy and radiotherapy.^59^ Many small-molecule inhibitors of Akt have been evaluated in clinical trials,^60^ but none have been approved for clinical use as of July 2022.

As a downstream effector of PI3K/Akt, mTOR is usually hyperactive in various tumor types. mTOR-selective or dual mTOR/PI3K small-molecule inhibitors^61^ have been developed, and four anticancer mTOR-specific inhibitors have been approved by the FDA: (1) sirolimus (rapamycin) to treat lymphangioleiomyomatosis^62^; (2) everolimus to treat advanced renal cell carcinoma (RCC), renal angiomyolipoma (AML), postmenopausal advanced HR-positive, HER2-negative breast cancer, progressive neuroendocrine tumors of pancreatic origin, and subependymal giant cell astrocytoma (SEGA)^63^; (3) temsirolimus to treat advanced RCC^64^; and (4) Fyarro (sirolimus albumin-bound nanoparticles, nab-sirolimus, ABI-009), the latest mTOR inhibitor approved in November 2021, to treat patients with locally advanced unresectable or metastatic malignant perivascular epithelioid cell tumor.^65^ PI3K/mTOR dual inhibitors, combining multiple therapeutic effects in a single molecule, reduce the activity of PI3K and mTOR by competitive interaction with the ATP-binding cleft of these enzymes. Recently, an array of inhibitors,^66–69^ such as dactolisib (BEZ235), samotolisib (LY3023414), and bimiralisib (PQR309), have been studied in clinical trials, but none has yet been approved for use clinically.

### Immunomodulatory signaling pathways

As newcomers to the human body, tumor cells generate and express specific antigens on their surface, which can be recognized and eliminated by immune cells such as cytotoxic T lymphocytes cells (CTLs). However, malignant tumor cells develop multiple escape mechanisms to evade immune recognition and surveillance. Reversing these immune evasion strategies is a promising approach for antitumor therapy. Immune checkpoint blockade (ICB) therapies targeting the programmed cell death protein 1 (PD1)/programmed cell death ligand 1 (PD-L1)^70,71^ pathway or cytotoxic T lymphocyte antigen 4 (CTLA4)^72–74^ have revolutionized the treatment landscape for multiple cancer types.

#### PD-1/PD-L1 signaling pathway

PD-1 is expressed on the surface of activated T cells and acts as an immunosuppressant, while its ligand PD-L1 is mainly overexpressed on tumor cells. The binding of PD-1 to PD-L1 suppresses T-cell-mediated immune responses by inhibiting T-cell proliferation, limiting cytokine production, and ultimately resulting in immune evasion of tumors.^75–77^

Blockade of the PD-1/PD-L1 interaction with specific antibodies results in the rescue of functionally exhausted T cells and the reactivation of immune responses. As of 2022, based on highly successful clinical trials, the FDA, European Medicines Agency (EMA), and National Medical Products Administration (NMPA) have approved 10 anti-PD-1 (nivolumab, pembrolizumab, cemiplimab, sintilimab, camrelizumab, toripalimab, tislelizumab, zimberelimab, prolgolimab, and dostarlimab) and three anti-PD-L1 antibodies (atezolizumab, durvalumab, and avelumab) for various hematological and solid malignancies.^78^

#### CTLA-4 signaling pathway

During the process of T-cell activation, membrane CTLA-4 and secreted soluble CTLA-4 are upregulated on CD8^+^ T cells and CD4^+^ T cells. Then, CD28 binds to the costimulatory molecules B7-1 (CD80) and B7-2 (CD86) as a secondary signal. CTLA-4 competitively binds to B7 to block the B7-CD28 signaling pathway.^79^ Moreover, the intracellular domain of CTLA-4 becomes phosphorylated, which generates a negative signal blocking the activation and function of T cells.^80^ In addition, CTLA-4 is abundantly found in forkhead box p3 (Foxp3)^+^ regulatory T cells (Tregs), which mainly works to suppress T-cell activity.^81^ Therefore, CTLA4 is considered a negative regulator of T-cell activation.

CTLA-4 inhibitors interfere with the interaction of CTLA-4 and B7 to erase the suppressive impact of CTLA-4 on T-cell activity and promote antitumor immune response, leading to tumor regression. Considerable evidence has also shown that CTLA-4 inhibitors function through antibody-dependent cell-mediated cytotoxicity (ADCC) via the Fc receptor (FcR) to remove Treg and downregulate the immunosuppressive effect effectively.^82^ Ipilimumab is a commonly used CTLA-4-blocking antibody approved by the FDA.^83^

**Fig. 1:**
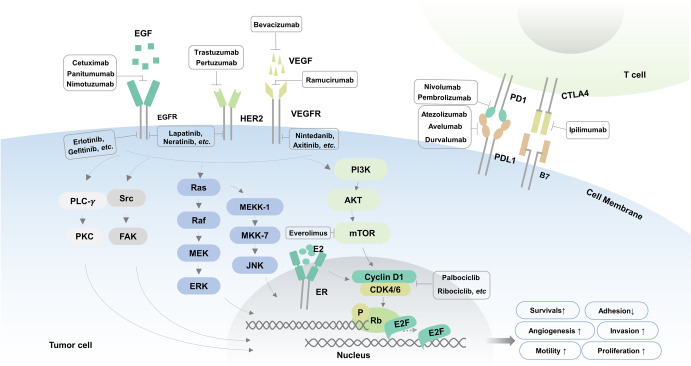
The pathway diagram of VEGFR, EGFR, HER2, CDK4/6, and PD1/PDL1 signaling. The signal transduction signaling pathways and some targeted inhibitors

The signaling pathways discussed above are deemed as potentials therapeutic targets. We summarized the main signal transduction pathways in Fig. 1 by illustrating key signal transduction processes. Besides, based on the defined signal pathways, these FDA-approved and commonly used targeted anti-cancer drugs were summarized in Table 1, as well as their related targets, indications, and categories (i.e., antibody or small-molecule inhibitors or ADCs).

**Table 1:** FDA-approved and commonly used targeted anti-cancer drugs

Drugs	Target	Category	Indications
Bevacizumab	VEGF	Antibody	1. Metastatic colorectal cancer: in combination with intravenous fluorouracil-based chemotherapy for first- or second-line treatment. 2. Metastatic colorectal cancer: in combination with fluoropyrimidine-irinotecan- or fluoropyrimidine oxaliplatin-based chemotherapy for second-line treatment in patients who have progressed on a first-line bevacizumab product-containing regimen. [Limitations of Use: Alymsys is not indicated for adjuvant treatment of colon cancer.] 3. Unresectable, locally advanced, recurrent or metastatic non-squamous non-small cell lung cancer, in combination with carboplatin and paclitaxel for first-line treatment. 4. Recurrent glioblastoma in adults. 5. Metastatic renal cell carcinoma in combination with interferon alfa. 6. Persistent, recurrent, or metastatic cervical cancer, in combination with paclitaxel and cisplatin, or paclitaxel and topotecan. 7. Epithelial ovarian, fallopian tube, or primary peritoneal cancer in combination with paclitaxel, pegylated liposomal doxorubicin, or topotecan for platinum-resistant recurrent disease who received no more than 2 prior chemotherapy regimens
Sorafenib	VEGFR, PDFGR, RAF, MEK, ERK	Small-molecule inhibitors	1. Unresectable hepatocellular carcinoma. 2. Advanced renal cell carcinoma. 3. Locally recurrent or metastatic, progressive, differentiated thyroid carcinoma refractory to radioactive iodine treatment
Regorafenib	VEGFR, PDGFR, Kit, REK, FGFR, Raf	Small-molecule inhibitors	1. Hepatocellular carcinoma who have been previously treated with sorafenib. 2. Metastatic colorectal cancer who have been previously treated with fluoropyrimidine-, oxaliplatin- and irinotecan-based chemotherapy, an antivegf therapy, and, if RAS wild-type, an anti-EGFR therapy. 3. Locally advanced, unresectable or metastatic gastrointestinal stromal tumor who have been previously treated with imatinib mesylate and sunitinib malate.
Donafenib	VEGFR, PDGFR, Raf	Small-molecule inhibitors	Have not received the whole body in the past inaccessibility of systematic treatment Patients with hepatocellular carcinoma
Anlotinib	VEGFR-1, VEGFR-2, VEGFR-3, c-KIT, PDGFR	Small-molecule inhibitors	1. For the treatment of patients with locally advanced or metastatic non-small cell lung cancer who have progressed or relapsed after receiving at least 2 prior systemic chemotherapies. 2. For patients with an EGFR mutation or positive for mesenchymal lymphoma kinase who have progressed after treatment with the appropriate standard targeted agent and have progressed or relapsed after at least 2 prior systemic chemotherapy regimens prior to initiation of therapy with this product. 3. For the treatment of patients with small cell lung cancer who have progressed or relapsed after at least 2 prior chemotherapy regimens.
Fruquinitinib	VEGFR1-3	Small-molecule inhibitors	Patients with metastatic colorectal cancer who have previously received fluorouracil, oxaliplatin and irinotecan-based chemotherapy, and who have previously received or are not suitable for VEGF treatment or EGFR treatment (RAS wild type)
Surufatinib	VEGFR1-3, CSF1R, FGFR1	Small-molecule inhibitors	Nonpancreatic neuroendocrine tumor with locally advanced or metastatic, progressive nonfunctional, well-differentiated (G1, G2) that cannot be resected surgically
Lenvatinib	VEGFR1-3, FGFR1-2, PDGFR, KIT, RET	Small-molecule inhibitors	1. For the treatment of patients with locally recurrent or metastatic, progressive, radioactive iodine-refractory differentiated thyroid cancer. 2. In combination with pembrolizumab, for the first-line treatment of adult patients with advanced renal cell carcinoma. 3. In combination with everolimus, for the treatment of adult patients with advanced renal cell carcinoma following one prior antiangiogenic therapy. 4. For the first-line treatment of patients with unresectable hepatocellular carcinoma. 5. In combination with pembrolizumab, for the treatment of patients with advanced endometrial carcinoma that is mismatch repair proficient (pmmr), as determined by an FDA-approved test, or not microsatellite instability-high (MSI-H), who have disease progression following prior systemic therapy in any setting and are not candidates for curative surgery or radiation.
Pazopanib	VEGFR1-3, PDGFR, c-KIT	Small-molecule inhibitors	1. Advanced renal cell carcinoma. 2. Advanced soft tissue sarcoma who have received prior chemotherapy.
Axitinib	VEGFR1-3, c-KIT, PDGF-R	Small-molecule inhibitors	1. In combination with avelumab, for the first-line treatment of patients with advanced renal cell carcinoma. 2. In combination with pembrolizumab, for the first-line treatment of patients with advanced RCC. 3. As a single agent, for the treatment of advanced renal cell carcinoma after failure of one prior systemic therapy.
Ramucirumab	VEGFR2	Antibody	1. As a single agent or in combination with paclitaxel, for treatment of advanced or metastatic gastric or gastro-esophageal junction adenocarcinoma with disease progression on or after prior fluoropyrimidine- or platinum-containing chemotherapy. 2. In combination with erlotinib, for first-line treatment of metastatic non-small cell lung cancer with egfr exon 19 deletions or exon 21 (l858r) mutations. 3. In combination with docetaxel, for treatment of metastatic non-small cell lung cancer with disease progression on or after platinum-based chemotherapy. Patients with EGFR or ALK genomic tumor aberrations should have disease progression on FDA-approved therapy for these aberrations prior to receiving cyramza. 4. In combination with Folfiri, for the treatment of metastatic colorectal cancer with disease progression on or after prior therapy with bevacizumab, oxaliplatin, and fluoropyrimidine. 5. As a single agent, for the treatment of hepatocellular carcinoma in patients who have an alpha-fetoprotein of ≥400 ng/ml and have been treated with sorafenib.
Apatinib	VEGFR-2	Small-molecule inhibitors	In combination with 1. Capecitabine for the treatment of patients with advanced or metastatic breast cancer whose tumors overexpress human HER2 and who have received prior therapy, including an anthracycline, a taxane, and trastuzumab. In combination with capecitabine. 2. Letrozole for the treatment of postmenopausal women with hormone receptor-positive metastatic breast cancer that overexpresses the HER2 receptor for whom hormonal therapy is indicated.
Panitumumab	EGFR	Antibody	For the treatment of wild-type RAS (defined as wild-type in both KRAS and NRAS as determined by an FDA-approved test for this use) metastatic colorectal cancer: 1. In combination with FOLFOX for first-line treatment. 2. As monotherapy following disease progression after prior treatment with fluoropyrimidine, oxaliplatin, and irinotecan-containing chemotherapy.
Cetuximab	EGFR	Antibody	1. *Head and neck cancer*: (1) Locally or regionally advanced squamous cell carcinoma of the head and neck in combination with radiation therapy. (2) Recurrent locoregional disease or metastatic squamous cell carcinoma of the head and neck in combination with platinum-based therapy with fluorouracil. (3) Recurrent or metastatic squamous cell carcinoma of the head and neck progressing after platinum-based therapy. 2. *Colorectal cancer*:(1) K-Ras wild-type, EGFR-expressing, metastatic colorectal cancer as determined by an FDA-approved test (1) in combination with FOLFIRI for first-line treatment (2) in combination with irinotecan in patients who are refractory to irinotecan-based chemotherapy (3) as a single-agent in patients who have failed oxaliplatin- and irinotecan-based chemotherapy or who are intolerant to irinotecan. [Limitations of Use: ERBITUX is not indicated for the treatment of Ras mutant colorectal cancer or when the results of the Ras mutation tests are unknown.] 3. *BRAF V600E mutation-positive metastatic colorectal cancer*: In combination with encorafenib, for the treatment of adult patients with metastatic colorectal cancer with a BRAF V600E mutation, as detected by an FDA-approved test, after prior therapy.
Nimotuzumab	EGFR	Antibody	In combination with radiotherapy for stage III/IV nasopharyngeal carcinoma with EGFR positive expression.
Gefitinib	EGFR	Small-molecule inhibitors	The first-line treatment of patients with metastatic non-small cell lung cancer whose tumors have EGFR exon 19 deletions or exon 21 (L858R) substitution mutations
Erlotinib	EGFR	Small-molecule inhibitors	1. For patients with metastatic non-small cell lung cancer whose tumors have EGFR exon 19 deletions or exon 21 (L858R) substitution mutations as detected by an FDA-approved test receiving first-line, maintenance, or second or greater line treatment after progression following at least one prior chemotherapy regimen. 2. First-line treatment of patients with locally advanced, unresectable or metastatic pancreatic cancer, in combination with gemcitabine.
Icotinib	EGFR	Small-molecule inhibitors	1. For the first-line treatment of patients with locally advanced or metastatic non-small cell lung cancer with sensitive mutations in the EGFR gene. 2. For the treatment of locally advanced or metastatic non-small cell lung cancer after failure of at least one prior chemotherapy regimen, which is primarily platinum-based combination chemotherapy. 3. For post-operative adjuvant therapy in stage II-IIIA with EGFR-sensitive mutations in non-small cell lung cancer. It is not recommended for use in patients with EGFR wild-type non-small cell lung cancer.
Dacomitinib	EGFR, HER1, HER2, HER4, DDR1, EPHA6	Small-molecule inhibitors	The first-line treatment of patients with metastatic non-small cell lung cancer with EGFR exon 19 deletion or exon 21 L858R substitution mutations as detected by an FDA-approved test.
Afatinib	EGFR, HER2, HER3	Small-molecule inhibitors	First-line treatment of patients with metastatic non-small cell lung cancer whose tumors have non-resistant EGFR mutations as detected by an FDA-approved test
Osimertinib	EGFR	Small-molecule inhibitors	1. As adjuvant therapy after tumor resection in adult patients with non-small cell lung cancer whose tumors have EGFR exon 19 deletions or exon 21 L858R mutations, as detected by an FDA-approved test. 2. The first-line treatment of adult patients with metastatic NSCLC whose tumors have EGFR exon 19 deletions or exon 21 L858R mutations, as detected by an FDA-approved test. 3. The treatment of adult patients with metastatic EGFR T790M mutation-positive NSCLC, as detected by an FDA-approved test, whose disease has progressed on or after EGFR TKI therapy.
Ametinib	EGFR	Small-molecule inhibitors	As a single agent for the treatment of BRAF-inhibitor treatment-naïve patients with unresectable or metastatic melanoma with BRAF V600E or V600K mutations as detected by an FDA-approved test
Furmonertinib	EGFR	Small-molecule inhibitors	1. Indicated for the first-line treatment of patients with locally advanced or metastatic non-small cell lung cancer with a sensitive mutation in the EGFR gene. 2. It is indicated as a single agent for the treatment of locally advanced or metastatic non-small cell lung cancer after failure of at least one prior chemotherapy regimen, primarily platinum-based combination chemotherapy.3. It is indicated as a single agent for the postoperative adjuvant treatment of stage II-IIIA non-small cell lung cancer with EGFR-sensitive mutations.4. It is not recommended for patients with EGFR wild-type non-small cell lung cancer.
Lapatinib	EGFR	Small-molecule inhibitors	1. Capecitabine for the treatment of patients with advanced or metastatic breast cancer whose tumors overexpress HER2 and who have received prior therapy, including an anthracycline, a taxane, and trastuzumab. 2. Letrozole for the treatment of postmenopausal women with hormone receptor-positive metastatic breast cancer that overexpresses the HER2 receptor for whom hormonal therapy is indicated.
Pyrotinib	EGFR, HER2	Small-molecule inhibitors	Combined with capecitabine, it is indicated for the treatment of patients with recurrent or metastatic breast cancer who are positive for HER2 and who have not received or has received trastuzumab in the past. Patients should receive chemotherapy with anthracyclines or taxanes before use.
Neratinib	EGFR, HER2, HER4	Small-molecule inhibitors	1. As a single agent, for the extended adjuvant treatment of adult patients with early-stage HER2-positive breast cancer, to follow adjuvant trastuzumab-based therapy. 2. In combination with capecitabine, for the treatment of adult patients with advanced or metastatic HER2-positive breast cancer who have received two or more prior anti-HER2-based regimens in the metastatic setting.
Trastuzumab	HER2	Small-molecule inhibitors	1. Adult patients with unresectable or metastatic HER2-positive breast cancer who have received a prior anti-HER2-based regimen either in the metastatic setting or in the neoadjuvant or adjuvant setting and have developed disease recurrence during or within six months of completing therapy. 2. Adult patients with unresectable or metastatic HER2-low (IHC 1+ or IHC 2+/ISH−) breast cancer, as determined by an FDA-approved test, who have received prior chemotherapy in the metastatic setting or developed disease recurrence during or within 6 months of completing adjuvant chemotherapy. 3. Adult patients with unresectable or metastatic non-small cell lung cancer (NSCLC) whose tumors have activating HER2 (ERBB2) mutations, as detected by an FDA-approved test, and who have received prior systemic therapy. 4. Adult patients with locally advanced or metastatic HER2-positive gastric or gastroesophageal junction adenocarcinoma who have received a prior trastuzumab-based regimen.
Pertuzumab	HER2	Small-molecule inhibitors	1. Use in combination with trastuzumab and docetaxel for the treatment of patients with HER2-positive metastatic breast cancer who have not received prior anti-HER2 therapy or chemotherapy for metastatic disease. 2. Use in combination with trastuzumab and chemotherapy as (1) neoadjuvant treatment of patients with HER2-positive, locally advanced, inflammatory, or early-stage breast cancer (either greater than 2 cm in diameter or node-positive) as part of a complete treatment regimen for early breast cancer. (2) adjuvant treatment of patients with HER2-positive early breast cancer at high risk of recurrence
Disitamab Vedotin	HER2	Antibody–drug conjugates	HER2 overexpression (2+ or 3+) in locally advanced or metastatic gastric cancer that has received at least two systems of chemotherapy
T-DM1 (trastuzumab Emtansine)	HER2	Antibody–drug conjugates	A single agent, for 1. The treatment of patients with HER2-positive, metastatic breast cancer who previously received trastuzumab and a taxane, separately or in combination. Patients should have either received prior therapy for metastatic disease, or developed disease recurrence during or within six months of completing adjuvant therapy. 2. The adjuvant treatment of patients with HER2-positive early breast cancer who have the residual invasive disease after neoadjuvant taxane and trastuzumab-based treatment.
Sacituzumab govitecan	TROP2	Antibody–drug conjugates	For the treatment of adult patients with 1. Unresectable locally advanced or metastatic triple-negative breast cancer who have received two or more prior systemic therapies, at least one of them for metastatic disease. 2. Locally advanced or metastatic urothelial cancer who have previously received platinum-containing chemotherapy and either PD-1 or PDL1 inhibitor
Palbociclib	CDK4/6	Small-molecule inhibitors	For the treatment of adult patients with HR-positive, HER2-negative advanced or metastatic breast cancer in combination with 1. An aromatase inhibitor as initial endocrine-based therapy in postmenopausal women or in men; or 2. Fulvestrant in patients with disease progression following endocrine therapy.
Ribociclib	CDK4/6	Small-molecule inhibitors	For the treatment of adult patients with HR-positive, HER2-negative advanced or metastatic breast cancer in combination with an aromatase inhibitor as initial endocrine-based therapy or fulvestrant as initial endocrine-based therapy or following disease progression on endocrine therapy in postmenopausal women or in men
Abemaciclib	CDK4/6	Small-molecule inhibitors	1. In combination with endocrine therapy (tamoxifen or an aromatase inhibitor) for the adjuvant treatment of adult patients with HR-positive, HER2-negative, node-positive, early breast cancer at high risk of recurrence and a Ki-67 score ≥20% as determined by an FDA approved test. 2. In combination with an aromatase inhibitor as initial endocrine-based therapy for the treatment of postmenopausal women, and men, with HR-positive, HER2-negative advanced or metastatic breast cancer. 3. In combination with fulvestrant for the treatment of adult patients with HR-positive, HER2-negative advanced or metastatic breast cancer with disease progression following endocrine therapy. 4. As monotherapy for the treatment of adult patients with HR-positive, HER2-negative advanced or metastatic breast cancer with disease progression following endocrine therapy and prior chemotherapy in the metastatic setting.
Pembrolizumab	PD-1	Antibody	1. Melanoma 2. Non-small cell lung cancer 3. Head and neck squamous cell cancer 4. Classical Hodgkin lymphoma 5. Primary mediastinal large b-cell lymphoma 6. Urothelial carcinoma 7. Microsatellite instability-high or mismatch repair deficient cancer 8. Microsatellite instability-high or mismatch repair deficient colorectal cancer 9. Gastric cancer 10. Esophageal cancer 11. Cervical cancer 12. Merkel cell carcinoma 13. Renal cell carcinoma 14. Endometrial carcinoma 15. Tumor mutational burden-high (tmb-h) cancer 16. Cutaneous squamous cell carcinoma 17. Triple-negative breast cancer
Nivolumab	PD-1	Antibody	1. Melanoma 2. Non-small cell lung cancer 3. Malignant pleural mesothelioma 4. Renal cell carcinoma 5. Classical Hodgkin lymphoma 6. Squamous cell carcinoma of the head and neck 7. Urothelial carcinoma 8. Colorectal cancer 9. Hepatocellular carcinoma 10. Esophageal cancer 11. Gastric cancer, gastroesophageal junction cancer, and esophageal adenocarcinoma
Atezolizumab	PD-1/PD-L1	Antibody	1. Urothelial carcinoma 2. Non-small cell lung cancer 3. Small cell lung cancer 4. Hepatocellular carcinoma 5. Melanoma
Durvalumab	PD-L1	Antibody	1. For the treatment of adult patients with unresectable, Stage III non-small cell lung cancer whose disease has not progressed following concurrent platinum-based chemotherapy and radiation therapy. 2. In combination with tremelimumab-actl and platinum-based chemotherapy, for the treatment of adult patients with metastatic non-small cell lung cancer with no sensitizing EGFR mutations or anaplastic lymphoma kinase genomic tumor aberrations. 3. In combination with etoposide and either carboplatin or cisplatin, as first-line treatment of adult patients with extensive-stage small cell lung cancer. 4. In combination with gemcitabine and cisplatin, as treatment of adult patients with locally advanced or metastatic biliary tract cancer. 5. In combination with tremelimumab-actl, for the treatment of adult patients with unresectable hepatocellular carcinoma.
Avelumab	PD-L1	Antibody	1. Merkel Cell Carcinoma (MCC): Adults and pediatric patients 12 years and older with metastatic MCC. 2. Urothelial Carcinoma (UC) (1) Maintenance treatment of patients with locally advanced or metastatic UC that has not progressed with first-line platinum-containing chemotherapy. (2) Patients with locally advanced or metastatic UC who: (1) Have disease progression during or following platinum-containing chemotherapy. (2) Have disease progression within 12 months of neoadjuvant or adjuvant treatment with platinum-containing chemotherapy. 3. Renal Cell Carcinoma (RCC): First-line treatment, in combination with axitinib, of patients with advanced RCC.
Ipilimumab	CTLA-4	Antibody	1. Melanoma 2. Renal cell carcinoma 3. Colorectal cancer 4. Hepatocellular carcinoma 5. Non-small cell lung cancer 6. Malignant pleural mesothelioma 7. Esophageal cancer
Crizotinib	ALK, HGFR(c-Met)、ROS1(c-cos) and RON	Small-molecule inhibitors	Patients with locally advanced or metastatic non-small cell lung cancer that is an ALK-positive as detected by an FDA-approved test
Ensartinib	ALK, EPHA2, c-MET	Small-molecule inhibitors	Treatment of ALK-positive patients with locally advanced or metastatic non-small cell lung cancer.
Ceritinib	ALK, IGF-1R, InsR, ROS1	Small-molecule inhibitors	Adults with metastatic non-small cell lung cancer whose tumors are ALK-positive as detected by an FDA-approved test.
Alectinib	ALK, RET	Small-molecule inhibitors	Patients with ALK-positive metastatic non-small cell lung cancer as detected by an FDA-approved test
Imatinib	BCR-ABL, C-Kit, PDGF	Small-molecule inhibitors	1. Newly diagnosed adult and pediatric patients with Philadelphia chromosome-positive chronic myeloid leukemia (Ph+ CML) in the chronic phase. 2. Patients with Philadelphia chromosome-positive chronic myeloid leukemia (Ph+ CML) in blast crisis (BC), accelerated phase (AP), or in chronic phase (CP) after the failure of interferon-alpha therapy. 3. Adult patients with relapsed or refractory Philadelphia chromosome-positive acute lymphoblastic leukemia (Ph+ ALL). 4. Pediatric patients with newly diagnosed Philadelphia chromosome-positive acute lymphoblastic leukemia (Ph+ ALL) in combination with chemotherapy. 5. Adult patients with myelodysplastic/myeloproliferative diseases(MDS/MPD) associated with platelet-derived growth factor receptor(PDGFR) gene re-arrangements. 6. Adult patients with aggressive systemic mastocytosis (ASM) without the D816V c-Kit mutation or with c-Kit mutational status unknown. 7. Adult patients with hypereosinophilic syndrome (HES) and/or chronic eosinophilic leukemia (CEL) who have the FIP1L1-PDGFRα fusion kinase (mutational analysis or fluorescence in situ hybridization [FISH] demonstration of CHIC2 allele deletion) and for patients with HES and/or CEL who are FIP1L1-PDGFRα fusion kinase negative or unknown. 8. Adult patients with unresectable, recurrent and/or metastatic dermatofibrosarcoma protuberans (DFSP). 9. Patients with Kit (CD117) positive unresectable and/or metastatic malignant gastrointestinal stromal tumors. 10. Adjuvant treatment of adult patients following resection of Kit (CD117) positive GIST.
Ripretinib	KIT, PDGFRA	Small-molecule inhibitors	Adult patients with advanced gastrointestinal stromal tumor who have received prior treatment with 3 or more kinase inhibitors, including imatinib.
Avapritinib	KIT, PDGFRA, CSFR1	Small-molecule inhibitors	1. Gastrointestinal Stromal Tumor (GIST): the treatment of adults with unresectable or metastatic GIST harboring a PDGFRA exon 18 mutation, including PDGFRA D842V mutations. 2. Advanced Systemic Mastocytosis (advsm): the treatment of adult patients with advsm. Advsm includes patients with aggressive systemic mastocytosis (ASM), systemic mastocytosis with an associated hematological neoplasm (SMAHN), and mast cell leukemia.
Sunitinib	KIT, PDGFRA, VEGFR, RET	Small-molecule inhibitors	1. Treatment of adult patients with gastrointestinal stromal tumor after disease progression on or intolerance to imatinib mesylate. 2. Treatment of adult patients with advanced renal cell carcinoma (RCC). 3. Adjuvant treatment of adult patients at high risk of recurrent RCC following nephrectomy. 4. Treatment of progressive, well-differentiated pancreatic neuroendocrine tumors in adult patients with unresectable locally advanced or metastatic disease.
Trametinib	MEK1/2	Small-molecule inhibitors	As a single agent for the treatment of BRAF-inhibitor treatment-naïve patients with unresectable or metastatic melanoma with BRAF V600E or V600K mutations. In combination with dabrafenib, for 1. The treatment of patients with unresectable or metastatic melanoma with BRAF V600E or V600K mutations. 2. The adjuvant treatment of patients with melanoma with BRAF V600E or V600K mutations and involvement of lymph node(s), following complete resection. 3. The treatment of patients with metastatic non-small cell lung cancer with BRAF V600E mutation. 4. The treatment of patients with metastatic non-small cell lung cancer (NSCLC) with BRAF V600E mutation as detected by an FDA-approved test. 5. The treatment of adult and pediatric patients 6 years of age and older with unresectable or metastatic solid tumors with BRAF V600E mutation who have progressed following prior treatment and have no satisfactory alternative treatment options.
Savolitinib	MET	Small-molecule inhibitors	Adult patients with locally advanced or metastatic non-small cell lung cancer with a mutation in exon 14 of the mesenchymal-epithelial transformation factor (MET) who have disease progression after platinum-containing chemotherapy or who are intolerant to standard platinum-containing chemotherapy
Everolimus	mTOR	Small-molecule inhibitors	1. The treatment of postmenopausal women with advanced hormone receptor-positive, HER2-negative breast cancer in combination with exemestane after failure of treatment with letrozole or anastrozole 2. Adults with progressive neuroendocrine tumors of pancreatic origin (PNET) and adults with progressive, well-differentiated, non-functional neuroendocrine tumors (NET) of gastrointestinal (GI) or lung origin that are unresectable, locally advanced, or metastatic 3. Adults with advanced renal cell carcinoma (RCC) after the failure of treatment with sunitinib or sorafenib. 4. Adults with advanced renal cell carcinoma (RCC) after the failure of treatment with sunitinib or sorafenib. 5. The treatment of adult and pediatric patients aged 1 year and older with TSC who have subependymal giant cell astrocytoma (SEGA) that requires therapeutic intervention but cannot be curatively resected. 6. The adjunctive treatment of adult and pediatric patients aged 2 years and older with TSC-associated partial-onset seizures.
Olaparib	PARP	Small-molecule inhibitors	1. Ovarian cancer: (1) for the maintenance treatment of adult patients with deleterious or suspected deleterious germline or somatic BRCA-mutated advanced epithelial ovarian, fallopian tube, or primary peritoneal cancer who are in complete or partial response to first-line platinum-based chemotherapy. Select patients for therapy based on an FDA-approved companion diagnostic for Lynparza. (2) in combination with bevacizumab for the maintenance treatment of adult patients with advanced epithelial ovarian, fallopian tube, or primary peritoneal cancer who are in complete or partial response to first-line platinum-based chemotherapy and whose cancer is associated with homologous recombination deficiency (HRD)-positive status defined by either a deleterious or suspected deleterious BRCA mutation, and/or genomic instability. Select patients for therapy based on an FDA-approved companion diagnostic for Lynparza. (3) for the maintenance treatment of adult patients with recurrent epithelial ovarian, fallopian tube, or primary peritoneal cancer, who are in complete or partial response to platinum-based chemotherapy. 2. Breast cancer: (1) for the adjuvant treatment of adult patients with deleterious or suspected deleterious gbrcam HER2-negative high-risk early breast cancer who have been treated with neoadjuvant or adjuvant chemotherapy. Select patients for therapy based on an FDA-approved companion diagnostic for Lynparza. (2) for the treatment of adult patients with deleterious or suspected deleterious gbrcam, HER2-negative metastatic breast cancer who have been treated with chemotherapy in the neoadjuvant, adjuvant, or metastatic setting. Patients with hormone receptor (HR)-positive breast cancer should have been treated with prior endocrine therapy or be considered inappropriate for endocrine therapy. Select patients for therapy based on an FDA-approved companion diagnostic for Lynparza. 3. Pancreatic cancer: for the maintenance treatment of adult patients with deleterious or suspected deleterious gbrcam metastatic pancreatic adenocarcinoma whose disease has not progressed on at least 16 weeks of a first-line platinum-based chemotherapy regimen. Select patients for therapy based on an FDA-approved companion diagnostic for Lynparza. 4. Prostate cancer: for the treatment of adult patients with deleterious or suspected deleterious germline or somatic homologous recombination repair (HRR) gene-mutated metastatic castration-resistant prostate cancer who have progressed following prior treatment with enzalutamide or abiraterone. Select patients for therapy based on an FDA-approved companion diagnostic for Lynparza.
Niraparib	PARP	Small-molecule inhibitors	1. For the maintenance treatment of adult patients with advanced epithelial ovarian, fallopian tube, or primary peritoneal cancer who are in complete or partial response to first-line platinum-based chemotherapy. 2. For the maintenance treatment of adult patients with recurrent epithelial ovarian, fallopian tube, or primary peritoneal cancer who are in complete or partial response to platinum-based chemotherapy.
Fluzoparib	PARP	Small-molecule inhibitors	For the treatment of platinum-sensitive recurrent ovarian cancer, tubal cancer, or primary peritoneal cancer patients with germ-line BRCA mutation (gbrcam) after second-line or above chemotherapy.
Pamiparib	PARP	Small-molecule inhibitors	For the treatment of patients with recurrent advanced ovarian cancer, tubal cancer, or primary peritoneal cancer with germ-line BRCA (gbrca) mutations who have received second-line or above chemotherapy.
Pralsetinib	RET	Small-molecule inhibitors	1. Adult patients with metastatic rearranged during transfection (RET) fusion-positive non-small cell lung cancer as detected by an FDA-approved test. 2. Adult and pediatric patients 12 years of age and older with advanced or metastatic RET-mutant medullary thyroid cancer who require systemic therapy. 3. Adult and pediatric patients 12 years of age and older with advanced or metastatic RET fusion-positive thyroid cancer who require systemic therapy and who are radioactive iodine-refractory (if radioactive iodine is appropriate).
Vemurafenib	BRAF V600E	Small-molecule inhibitors	1. The treatment of patients with unresectable or metastatic melanoma with BRAF V600E mutation. 2. The treatment of patients with Erdheim-Chester Disease with BRAF V600 mutation.
Dabrafenib	BRAF V600E	Small-molecule inhibitors	1. As a single agent for the treatment of patients with unresectable or metastatic melanoma with BRAF V600E mutation. 2. In combination with trametinib, for:1. The treatment of patients with unresectable or metastatic melanoma with BRAF V600E or V600K mutations. 3. The adjuvant treatment of patients with melanoma with BRAF V600E or V600K mutations and involvement of lymph node(s), following complete resection. 4. The treatment of patients with metastatic non-small cell lung cancer with BRAF V600E mutation. 5. The treatment of patients with locally advanced or metastatic anaplastic thyroid cancer (ATC) with BRAF V600E mutation and with no satisfactory locoregional treatment options. 6. The treatment of adult and pediatric patients 6 years of age and older with unresectable or metastatic solid tumors with BRAF V600E mutation who have progressed following prior treatment and have no satisfactory alternative treatment options.
Abiraterone	CYP17	Small-molecule inhibitors	1. Metastatic castration-resistant prostate cancer. 2. Metastatic high-risk castration-sensitive prostate cancer.

## Molecular imaging

Molecular imaging is a noninvasive medical imaging method that enables the visualization, characterization, and measurement of biological processes at the molecular and cellular levels in tumors.^84,85^ In contrast to conventional imaging modalities that primarily image differences in the structure of tissues or organs, molecular imaging reveals the physiological activities or expression status of specific molecules within a tissue or organ by employing medical imaging modalities with or without tracers.

**Fig. 2:**
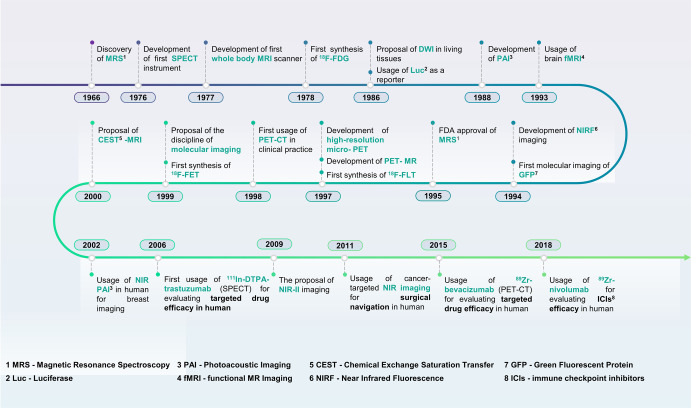
The historic steps in molecular imaging technology

From the phenomenon of magnetic resonance spectroscopy observed in 1966,^86^ to the first SPECT instrument developed in 1976,^87^ to the first whole-body MRI scanner in 1977,^88^ to luciferase (Luc) used as a reporter of gene expression in vivo in 1986,^89^ to near-infrared fluorescence (NIRF) imaging developed in 1994,^90^ to the first PET-CT completing the unity of both function and anatomical imaging in clinical practice in 1998,^91^ to the photoacoustic imaging first used in human in 2002,^92^ to the NIR-II imaging proposed in 2009.^93^ Some other historic steps promote the development of molecular imaging (Fig. 2). Given this excellent work, in September 1999, Weissleder and other imaging authorities held an international imaging conference in Jackson, the capital of Mississippi. The participating experts agreed that molecular imaging has emerged as a new field. Since then, molecular imaging has accelerated oncology detection, surgical guidance, targeted drug delivery, imaging-guided therapy, and efficacy evaluation.^94^ Given that several valuable reviews on molecular imaging have recently been published,^95–100^ here we only highlight the modalities most commonly used and their application for evaluating the efficacy of cancer-targeted therapy.

### Nuclear imaging

#### PET imaging

PET is a molecular imaging technique that uses radiotracers to visualize and quantify the biological characteristics of tumors. PET is based on the principle that radionuclides emit positrons when decaying. The emitted positron is annihilated with an electron to create two 511 keV gamma rays at an angle of 180°.^101^ A ring of detectors is used to detect these emitted gamma rays. Radionuclides that are available for PET imaging in clinical and research applications include fluorine-18 [^18^F], carbon-11 [^11^C], zirconium-89 [^89^Zr], gallium-68 [^68^Ga], and copper-64 [^64^Cu]. Among these, ^18^F is most commonly used for clinical applications because of its beneficial half-life (*T*_1/2_ = 1.8 h), positron yield, and associated detection sensitivity.^102^

Owing to the development of nuclear medicine, there is now a wide variety of radiopharmaceuticals available in clinical practice to evaluate the biological features of tumors, such as ^18^F-FDG for tumor metabolism,^101^ ^18^F-FMISO for hypoxia,^103^ ^18^F-FLT for tumor cell proliferation,^104^ ^18^F-labeled amino acids for protein synthesis,^105^ and ^15^O–water for blood flow.^106^ Furthermore, PET, in combination with tumor-specific monoclonal antibodies (immune-PET), has broadened the application of PET imaging. Various monoclonal antibodies and radionuclides have been explored to develop immune-PET tracers.^107^ In addition, peptides and other receptor-targeting compounds, such as nanobodies or bispecific antibodies, are being used to design novel immune-PET tracers.^107,108^ By dynamically monitoring tumor antigens’ expression, immune-PET imaging is a promising technique for evaluating the efficacy of cancer-targeted therapy.

Inherent advantages of PET imaging include its high sensitivity and quantifiable imaging parameters, such as standardized uptake value (SUV). PET-CT combining anatomical and functional imaging information becomes possible to assess the molecular features of tumors with highly accurate anatomical structure correction. Furthermore, PET–MRI combining PET with MRI extends the scope of multimodality imaging and reduces patients’ exposure to radiation.^109^

#### SPECT imaging

SPECT is another nuclear imaging modality that uses radionuclides that emit single photons, such as technetium-99m (^99m^Tc), iodine-123 (^123^I), and indium-111 (^111^In). The emitted single photons are subsequently detected by a gamma camera to image the organs of interest or the whole body. SPECT has limited spatial resolution and lower sensitivity in tumor detection compared to PET. The quantification of SPECT is also more challenging. However, despite these limitations, it is more commonly used than PET in clinical practice. This is mainly due to its advantage of having a large number of radiopharmaceuticals that are readily available for clinical use. Specifically, ~85% of radiopharmaceuticals can be detected in clinical practice by SPECT imaging.^110^ Tumor-specific biological compounds, such as antibodies or peptides, can be used for radiolabeling to produce tumor-targeted SPECT radiopharmaceuticals, e.g., ^123^I-VEGF for VEGFR targeting, ^111^In-bevacizumab for VEGF targeting, ^111^In-trastuzumab for HER2 targeting, and ^111^In-EGF for EGFR targeting.^111–113^ These approaches have been explored for monitoring the responses to anticancer treatment in humans.^110,111^

#### Magnetic resonance imaging

MRI is a noninvasive imaging technique often classified as an anatomical imaging modality. When placed in a strong magnetic field, specific atomic nuclei can absorb radiofrequency energy and align like small magnets because of their spin. The absorption of energy by the nuclei causes a transition from a high- to a low-energy state. This further induces a voltage that can be detected, amplified, and displayed as “free-induction decay (FID),” which can be resolved by a mathematical process to generate high-resolution anatomical images.^114^ Moreover, using specific techniques, MRI demonstrates the ability to image molecular processes within a tumor. This provides functional information on tumor vascular permeability, perfusion,^115^ vascular volume and flow, tortuosity of extracellular space,^115^ and hypoxia.^116^ As such, these MRI techniques can be classified as forms of functional imaging.

### Dynamic contrast-enhanced MRI

As suggested by its name, dynamic contrast-enhanced MRI (DCE-MRI) demonstrates the temporal enhancement pattern of a tissue following the injection of a paramagnetic contrast agent (CA), such as gadolinium-diethylenetriamine pentaacetic acid (Gd-DTPA). Factors influencing the tumor uptake of CA include blood perfusion, tissue vascularization, vessel permeability, cell density, extravascular extracellular volume fraction, and extracellular matrix density.^117^ A CA has two important physicochemical properties, namely, the relaxation effect and the susceptibility effect. MRI sequences studying the relaxation effect are termed DCE-MRI or *T1-W DCE*, while those assessing the susceptibility effect are termed dynamic susceptibility contrast (DSC)-MRI or T2*-W DCE.

*T1-W DCE* is sensitive to the presence of CA in the extravascular extracellular space. Tofts’ standard pharmacokinetic model is the mathematical model most commonly used in clinical studies for analyzing human DCE-MRI data.^118^ Tofts’ model introduces three key parameters, namely, *K*^trans^, *V*_e_, and *V*_p_.^119,120^ *K*^trans^ is the bulk transfer coefficient, which reflects the leakage of contrast from the vascular to the extravascular compartment.^119^ *V*_e_ is the fractional volume of the extravascular extracellular space (EES),^119^ while *V*_p_ represents the concentration of CA in plasma space.^120^ The efflux rate constant from EES to plasma (*K*_ep_), which is the ratio of *K*^trans^ to *V*_e_, is also frequently described in DCE-MRI.^120^ DCE-MRI has been used to evaluate the treatment response and demonstrate prognostic value in patients receiving cancer-targeted therapy. This is elaborated on in the following section.

T2*-W DCE is sensitive to the vascular phase of CA and is used to evaluate tissue perfusion and blood volume. The following parameters are measured in T2*-W DCE: (1) regional blood volume (rBV), which is defined as the volume (ml) of blood perfusing vessels in a voxel divided by the tissue mass in that voxel (g); (2) mean transit time (MTT), which is the average transit time of a CA particle through the capillary bed; and (3) regional blood flow (rBF), which reflects the tissue perfusion and is measured in milliliters per minute.

### Diffusion-weighted imaging

Diffusion-weighted imaging (DWI) is an MRI technique that generates signal contrast based on differences in the diffusion of water molecules. Within the body, water is distributed in the intracellular and extracellular compartments. The water molecules in the extracellular compartments diffuse relatively freely, while those within the intracellular compartments experience more restricted diffusion. The diffusion of water molecules can be quantitatively evaluated by the apparent diffusion coefficient (ADC). A lower ADC value reflects restricted diffusion. In tumor tissue, especially those with a high histological grade, the ADC value is lower than that in surrounding normal tissue. This is due to the high cellularity of tumors, as cellularity is positively correlated with the degree to which the diffusion of water is restricted.^121–124^ The use of DWI for evaluating the response to anticancer treatments has been explored. An increase in the ADC value can be observed at an earlier stage than a decrease in tumor size.^125^

### Magnetic resonance spectroscopy

Magnetic resonance spectroscopy (MRS) uses the same principles of signal acquisition as other MRI techniques. MRS studies in a medical context usually involve the detection of radiofrequency electromagnetic signals that are produced by chemical compounds. As a result, this technique provides chemical information on tissue metabolites.^126^ Therefore, MRS can be used to monitor the metabolic variations caused by treatments and treatment efficacy.^127^

### Chemical exchange saturation transfer

Chemical exchange saturation transfer (CEST) is an advanced MRI technique in which the exchangeable protons on target metabolites are selectively saturated and exchanged with water protons. The relative concentration of target metabolites can be measured with enhanced sensitivity by detecting the attenuation of the water proton signal indirectly.^112^ Amide proton transfer (APT) imaging is the CEST technique most commonly used in a clinical context to indirectly detect proteins and peptides in tissue. This technique provides important information for the diagnosis and monitoring of tumors.

### Targeted magnetic resonance imaging

Like other tumor-targeted imaging modalities, targeted magnetic resonance imaging (TMRI) can visualize tumor-specific molecular markers with targeted magnetic nanoparticles. This provides the opportunity to classify patients, deliver individualized therapy directly to tumors, and monitor the treatment response through MRI for tumors expressing specific biomarkers.^128,129^

### Optical imaging

Optical imaging is a noninvasive technique that uses light and optical properties of protons to image tissues, cells, and even molecules within the body.^130^ One of the major advantages of optical imaging is that it uses nonionizing radiation, making it much safer than techniques that use ionizing radiation such as X-rays. This makes optical imaging suitable for being repeatedly used to monitor gene expression, disease progression, or treatment response. Optical imaging includes multiple submodalities, such as bioluminescence imaging (BLI), chemiluminescence, Cherenkov imaging, and fluorescence imaging (FLI).^131^

Bioluminescence imaging makes use of the reaction between luciferases and their substrates to produce light.^131^ Luciferases, such as firefly luciferase, can be constitutively or inducibly expressed, and as such used for tracking the expression of targeted genes or monitoring tumor growth or regression to evaluate drug efficacy. These applications of BLI have been routinely used in preclinical studies.^132–135^ However, the drawbacks of BLI, such as the need for cell transfection and administration of a reactive substrate, prevent its clinical translation.

Fluorescence imaging provides images of tumors by detecting the emitted light that is generated from genetically encoded fluorescent proteins or fluorescent dyes after excitation by light of a different wavelength.^131^ The fluorescent dyes can be used to label tumor-specific antibodies, peptides, or nanobodies, enabling tumor-targeted molecular imaging. In comparison with BLI, a significant disadvantage of FLI is that its signal-to-background ratio is lower due to the auto-fluorescent noise coming from endogenous fluorophores within the tissue, which absorbs the excitation light. Other fundamental factors influencing the image quality of FLI are diffraction and diffusion.^131^ Diffraction reduces the spatial resolution of fluorescent images, while diffusion is caused by the tissue scattering of light, which limits the tissue penetration depth. Near-infrared (NIR) fluorescence imaging, including the first NIR (NIR-I) window (650–950 nm) and second NIR (NIR-II) window (1000–1700 nm), was emerged recently as an attractive imaging modality with high sensitivity, relative safety, and low cost. Compared with traditional NIR-I imaging, NIR-II imaging has less autofluorescence, absorption and scattering of light, higher penetration depths, and spatiotemporal resolution for biological tissues.^136^ FLI is primarily used in preclinical studies for tumor detection, fluorescence image-guided surgery, and monitoring of response to therapy.^137–140^ Along with the development of good manufacturing practice (GMP) tracers and clinical imaging systems, NIR FLI has been actively explored in early clinical trials for guiding cancer surgeries.^141–144^ However, the clinical application of FLI for evaluating the efficacy of targeted treatment has not been reported.

### Photoacoustic imaging

Photoacoustic imaging (PAI) is a novel noninvasive molecular imaging modality, which generates an ultrasound signal based on the photoacoustic effect. When laser pulses are delivered to a material, some of the energy is absorbed and converted to heat, resulting in a thermoelastic expansion that generates an ultrasonic signal from which images can be produced.^145^ As a hybrid of optical imaging and ultrasound imaging, PAI combines the high contrast and sensitivity of the optical property and high ultrasonic spatial resolution in a single imaging modality. In addition, the unprecedented imaging depth (up to centimeters) makes this a promising technique for various clinical applications.^146,147^ PAI can be used to analyze various endogenous contrast agents such as oxygenated and deoxygenated hemoglobin, lipids, melanin, and water.^147^ By using multiwavelength measurement, PAI can simultaneously quantify the concentrations of these endogenous chromophores and further provide biological information on tissues that reflects their different physiological or pathophysiological status.^148^ Furthermore, by using tumor-specific exogenous contrast agents, PAI can identify tumor cells and monitor the expression of tumor-specific biomarkers.^149–153^ Examples of these agents include gold nanoparticle-conjugated peptides or antibodies for EGFR and HER2 molecular imaging.^152,154^ The ability to perform both functional (by endogenous contrast) and molecular (by exogenous contrast) imaging makes PAI an attractive technique for evaluating tumor-targeted therapy.

### Ultrasound imaging

Ultrasound is a technique that uses high-frequency sound waves to produce anatomical images. It possesses several advantages, such as high availability, lack of radioactivity, and cost-effectiveness. These merits make it suitable to be repeatedly used in clinical practice. The Doppler technique allows ultrasound to be used to assess the blood flow in tumors. More importantly, with the use of contrast agents such as microbubbles, dynamic contrast-enhanced ultrasound (DCE-US) can measure longitudinal changes in hemodynamic parameters (e.g., perfusion, flow velocity) and morphological parameters (e.g., blood volume, vascular heterogeneity) of a given tumor relative to the findings in a pretreatment baseline assessment. The value of information on the changes in these parameters for monitoring the therapeutic response induced by anti-angiogenic therapies has been explored.^155^

### Molecular imaging probes

Among the above-mentioned imaging modalities, some modalities, such as PET, SPECT, and optical imaging, require the injection of imaging probes into the studied subjects to acquire an imaging signal. On the other hand, other modalities, such as photoacoustic imaging and MRI, can monitor the biological change associated with diseases either through the injection of exogenous molecular probes or by using endogenous molecules.^156^ Tumor nonspecific imaging probes, such as ^18^F-FDG for PET imaging and ^99m^Tc-sulfur colloid for SPECT imaging, have been widely used in clinical practice for detecting lesions or lymph nodes in cancer patients and for evaluating the efficacy of anticancer treatments.^157–159^ However, concerning tumor-specific imaging probes, many are still under development and are being tested in preclinical or early-stage clinical studies.^160–162^ A comprehensive review of all molecular imaging probes is beyond the scope of this paper. Therefore, here we only briefly describe the tumor-specific imaging probes that have been employed to evaluate the efficacy of cancer-targeted therapy. The mechanism of action of tumor-specific imaging probes is based on the concept that a carrier molecule that is labeled with a positron emitter for PET imaging, a single-photon emitter for SPECT imaging, or a fluorophore for fluorescent imaging specifically binds to a certain tumor target. Carrier molecules can be monoclonal antibodies, monoclonal antibody fragments, affibody molecules, small peptides, or small molecules that specifically target certain cell surface markers that are overexpressed in tumors.^163–165^ Moreover, carrier molecules can also be small molecules that detect the acidic microenvironment of tumors.^142^

### Monoclonal antibody

Monoclonal antibody (mAb)-based probes have been most commonly studied in evaluations of cancer-targeted therapy using molecular imaging, mainly due to their high specificity and high binding affinity. In addition, the relative stability and tolerance of chemical modifications to mAbs make them desirable for creating moieties targeting radionuclides or fluorophores. For PET and SPECT imaging, owing to the prolonged circulation time of mAbs in the body, radionuclides with long physical half-lives, such as ^124^I (*t*_½_ = 100.3 h),^166^ ^89^Zr (*t*_½_ = 78.4 h),^166^ and ^111^In (*t*^½^ = 2.8 days),^167^ should be chosen for radiolabeling. PET imaging of ^89^Zr-mAb has shown it to be a biomarker for predicting the efficacy of cancer-targeted treatments in xenograft models and early clinical trials.^163,168^ Nevertheless, the potential of mAb PET imaging or fluorescent imaging is limited by the slow clearance of intact antibodies from the blood, which causes undesirable high background signals and excessive nonspecific tissue accumulation such as in the liver.^169^

### Monoclonal antibody fragments

To reduce the undesirable high background signal and nonspecific tissue accumulation of mAb probes, specifically in PET and SPECT imaging, there is a demand for the use of antibody fragments that are cleared more rapidly from circulation. Furthermore, the shorter circulation time of antibody fragments requires shorter-lasting radionuclides such as ^68^Ga (*t*_½_ = 68 min),^170^ ^99m^Tc (*t*_½_ = 6 h),^167^ and ^64^Cu (*t*_½_ = 12.7 h) for radiolabeling, which in turn decreases radiation exposure in patients. All of these advantages make antibody fragments an attractive alternative to employ for molecular imaging.

Various antibody-derived fragments with different sizes, serum half-lives, bio-distributions, and levels of tumor penetration have been developed.^169^ Full-length antibodies can be digested by enzymes to produce F(ab’)_2_ (110 kDa) and F(ab) (50 kDa) fragments, or genetically engineered to generate a variety of antibody derivatives such as minibodies (75 kDa), scFvs (26 kDa), diabodies (50 kDa), and nanobodies (12–15 kDa).^167,169^ These antibody fragments retain the specificity of binding to the molecular targets of their parental IgG. However, much of the data on their application in molecular imaging has been obtained from preclinical studies.^167^ Clinical translational studies on them have been performed in only limited numbers of cancer types and patients.^167^ Examples include the use of a ^68^Ga-labeled anti-HER2 VHH for detecting lesions in patients with breast cancer.^171^

### Affibody molecules

Affibody molecules are engineered scaffold proteins consisting of 58 amino acids with a molecular weight of 6–7 kDa, which meet the requirements for an optimal balance between clearance from circulation and extravasation.^172^ This ensures the high absolute tumor uptake of affibody molecules and further increases the signal-to-background ratio. Several affibody molecules with high affinity to VEGFR2, EGFR, HER2, HER3, and IGF-1R have been demonstrated as probes for radionuclide or fluorescent molecular imaging in preclinical settings.^167,173^ Clinical evaluation of radiolabeled affibody molecules has been explored for HER2 imaging^174–177^ and fluorescent dye-labeled affibody molecules (ABY-029) for EGFR imaging have been tested in patients with recurrent glioma.^178^

### Peptides

According to the definition by the United States Food and Drug Administration (FDA), peptides are proteins consisting of fewer than 40 amino acids. Although their binding affinity is lower than that of antibodies, they are small, easy to synthesize, and capable of flexible chemical modification.^179^ With these distinctive advantages over antibodies, peptides have been increasingly used as probes for tumor receptor imaging, such as peptides for PD-L1 imaging,^180–183^ integrin αvβ3 imaging,^184–186^ and somatostatin receptor imaging.^187^ To date, many peptide-based probes have been developed and clinically used for cancer diagnosis and treatment.^187–189^

## Application of molecular imaging in targeted therapy

**Fig. 3:**
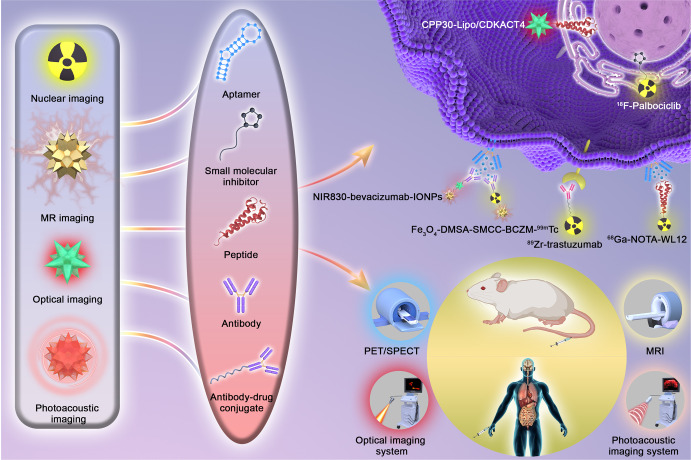
The diagram of molecular imaging and functional imaging in cancer-targeted therapy. The probes are constructed by imageable agent and their targets, respectively. On the cellular level, probes can bind to cell surface receptors or targets in the cytoplasm or nucleus to visualize and measure the target. After the probes are injected into animals or the human body, quantitative measures of probe uptake are used as predictive or evaluative assays for response to targeted therapy by different molecular imaging technologies

As discussed above, both conventional imaging modalities and standard-of-care assessment of tumor responses to treatment are based on morphological indicators such as tumor size. Molecular imaging is more sensitive and may detect changes earlier than morphological changes in tumors because it can visualize the expression of a specific molecule when used to monitor therapeutic efficacy.^190^ Figure 3 shows the representative diagram of how molecular imaging probes are designed and how responses to targeted therapy are evaluated.

### Nuclear imaging

Because of temporal and spatial heterogeneity and discordance in gene expression status between primary and distant metastatic lesions,^191^ the reproducible, noninvasive, whole-body evaluation of the efficacy of targeted therapy is critical for determining optimal treatment options. Among various types of molecular imaging, radionuclide imaging is advantageous for clinical usage because of the excellent sensitivity and tissue penetration of radionuclides. This approach would facilitate treatment optimization, prevent useless prescriptions, avoid unnecessary side effects, and, more importantly, prevent treatment failure in nonresponding patients.

Although nuclear imaging has been mainly utilized for monitoring the pharmacokinetics and pharmacodynamics of targeted drugs noninvasively,^192^ a discussion of this is outside the scope of this review. We instead discuss the application of nuclear imaging, including PET/CT and SPECT, in evaluating the response and predicting the prognosis after targeted therapy, among which the clinical application is summarized in Table 2.

**Table 2:** Clinical application of nuclear imaging in cancer-targeted therapy

Technique	Probe	Drug	Targeted signaling pathway	Tumor	*N*	Results	References
PET	^18^F-FDG	Bevacizumab and irinotecan	VEGF	Recurrent high-grade glioma	25	In multivariate analysis, the SUV_max_ and the T:CL ratio were the most powerful independent predictors of PFS (*P* = 0.001, HR = 8.41; *P* = 0.004; HR = 4.56, respectively) and OS (*P* = 0.038, HR = 3.28; *P* = 0.001, HR = 5.96, respectively) among all variables tested: the histological grade, KPS, corticotherapy, and the number of previous treatments. Sensitivity and specificity for relapse at 6 months were 66.7% and 100%, respectively, for the SUV_max_ and 61.9% and 100%, respectively, for the T:CL ratio.	^278^PMID: 22379188
PET	^18^F-FDG	Bevacizumab	VEGF	Metastatic colorectal cancer (mCRC)	19	In the group of radiological responders, the median baseline SUV_max_ was 3.77 [interquartile range (IQR): 2.88–5.60] compared with 7.20 (IQR: 4.67–8.73) in nonresponders (*P* = 0.021). A higher follow-up SUV_max_ was correlated with worse PFS (*P* = 0.012). Progression-free survival was significantly shorter in patients with a measurement of microvessel density (MVD) > 10 than in patients with lower MVD (10 months compared with 16 months, *P* = 0.016).	^277^PMID: 22596235
PET	^18^F-FDG	Sunitinib, sorafenib, or pazopanib	VEGF	Metastatic renal cell carcinoma (mRCC)	56	MTV and TLG could provide additional prognostic information in patients with clinically high-risk metastatic RCC treated with anti-vascular endothelial growth factor-targeted therapies.	^276^PMID: 28288043
PET	^18^F-FDG	Bevacizumab	VEGF	Colorectal cancer with liver metastases	7	Complete response (CR) was evident on FDG-PET in 10/17 (58%) lesions, whereas only 4/17 (23%) were deemed to have CR by CT. Similarly, only 1 of 17 (6%) lesions appeared stable by FDG-PET criteria, whereas 3 (18%) were classified as stable disease (SD) according to the size of the CT. FDG-PET findings correlated better than CT with pathology and were more indicative of pathology.	^201^PMID: 16417400
PET	^18^F-FDG	Bevacizumab	VEGF	High-risk locally advanced rectal cancer	61	Early total-lesion glycolysis and its percentage change compared with baseline (ΔTLG-early) could discriminate TRG1 from TRG2–TRG5. Only receiver-operating-characteristic analysis of ΔTLG-early showed an area under the curve >0.7 (0.76), with an optimal cut-off at 59.5% (80% sensitivity, 71.4% specificity), for identifying TRG1. Late metabolic assessment could not discriminate between the two groups. After a median follow-up of 98 months (range, 77–132 months), metabolic responders (ΔTLG-early ≥ 59.5%) demonstrated significantly higher 10-year progression-free survival (89.3% vs. 63.6%, *P* = 0.02) and cancer-specific survival (92.9% vs. 72.6%, *P* = 0.04) than incomplete metabolic responders.	^202^PMID:30877175
PET	^18^F-FDG ^18^F-FLT	Regorafenib	VEGF	Metastatic colorectal cancer refractory to all standard therapies	61	Five responders (8.2%) and 13 nonresponders (21.3%) met the CT and ^18^F-FLT PET/CT criteria (maximum standardized uptake value decrease ≥10.6% for responders). Forty-three (70.5%) exhibited discordant responses on CT and ^18^F-FLT PET/CT (McNemar test, *P* < 0.001). Comparison of PFS and OS according to ^18^F-FLT PET/CT response revealed slightly longer PFS (*P* = 0.015) in responders, but the correlation with OS was not significant. The PET Response Criteria in Solid Tumors (PERCIST) of ^18^F-FDG PET/CT revealed differences in PFS and OS between partial metabolic response (PMR) and non-PMR (*P* = 0.048 and *P* = 0.014, respectively), and between progressive metabolic disease (PMD) and non-PMD (*P* = 0.189 and *P* = 0.007, respectively).	^280^PMID: 31041456
PET	^18^F-FLT	Bevacizumab and irinotecan	VEGF	Recurrent malignant gliomas	21	Metabolic responders survived three times as long as nonresponders (10.8 vs. 3.4 months; *P* = 0.003) and tended to have prolonged progression-free survival (*P* = 0.061). Both early and later FLT-PET responses were more significant predictors of overall survival (1–2 weeks, *P* = 0.006; 6 weeks, *P* = 0.002), compared with the MRI responses (*P* = 0.060 for both 6-week and best responses).	^360^PMID: 17947718
PET	^18^F-FLT	Bevacizumab	VEGF	Recurrent malignant glioma	30	Early and late changes in tumor ^18^F-FLT uptake were more predictive of overall survival than MRI criteria (*P* < 0.001 and *P* = 0.01, respectively). ^18^F-FLT uptake changes were also predictive of progression-free survival (*P* < 0.001).	^361^PMID:22159180
PET	^18^F-FLT	Gefitinib	VEGF	Advanced adenocarcinoma of the lung	28	Pretreatment SUV_max_ of the tumors did not differ between responders and nonresponders. At 7 days after the initiation of therapy, the percent changes in SUV_max_ were significantly different (−36.0 ± 15.4% vs. 10.1 ± 19.5%; *P* < 0.001). Decrease of > 10.9% in SUV_max_ was used as the criterion for predicting response. The positive and negative predictive values were both 92.9%. The time to progression was significantly longer in FLT-PET responders than in nonresponders (median, 7.9 vs. 1.2 months; *P* = 0.0041).	^362^PMID: 19010859
PET	^89^Zr-bevacizumab	Everolimus	VEGF	Metastatic renal cell carcinoma (mRCC)	13	After 2 weeks of everolimus, median SUV_max_ was 6.3 (1.7–62.3), corresponding to a mean decrease of 9.1% (*P* < 0.0001). At the 6th week, a mean decrease in SUV_max_ of 23.4% compared with baseline was found in 70 evaluable lesions of 10 patients, with a median SUV_max_ of 5.4 (1.1–49.4, *P* < 0.0001). All 10 patients who continued treatment had stable disease in the third month.	^225^PMID: 28082434 (2017)
PET	^89^Zr-bevacizumab	Bevacizumab/interferon-α Sunitinib	VEGF	Metastatic renal cell carcinoma	22	Bevacizumab/interferon-α induced a mean change in tumor SUV_max_ of −47.0% (range, −84.7% to +20.0%; *P* < 0.0001) at 2 weeks and an additional −9.7% (range, −44.8% to +38.9%; *P* = 0.015) at 6 weeks. In the sunitinib group, the mean change in tumor SUV_max_ was −14.3% at 2 weeks (range, −80.4% to +269.9%; *P* = 0.006), but at 6 weeks the mean change in tumor SUV_max_ was +72.6% (range, −46.4% to +236%; *P* < 0.0001) above baseline. A baseline mean tumor SUV_max_ > 10.0 in the three most intense lesions corresponded with a longer time to disease progression (89.7 vs. 23.0 weeks; hazard ratio, 0.22; 95% confidence interval, 0.05–1.00).	^226^PMID: 25476536 (2015)
SPECT	^111^In-bevacizumab	Sorafenib	VEGF	Clear cell renal cell cancer (ccRCC)	14	Treatment with sorafenib resulted in a significant decrease of ^111^In-bevacizumab uptake in the tumor in patients with ccRCC (mean change, −60.5%; range, +1.5% to −90.1%).	^111^PMID: 20956472 (2010)
PET/CT	^18^F-FDG	Cetuximab	EGFR	Metastatic colorectal cancer (mCRC) with wild-type K-ras	27	Early response evaluation by ^18^F-FDG PET/CT predicts (*P* = 0.001) and OS (*P* < 0.001) at the end of the first week in patients with mCRC receiving third-line cetuximab-based therapy.	^271^PMID: 25608159
PET	^18^F-FDG	Trastuzumab	HER2	HER2-positive inoperable, locally advanced, recurrent, or metastatic gastric cancer	124	Among HER2-positive gastric cancer patients treated with trastuzumab, patients with WB TLG > 600 (HR 2.703; *P* = 0.026) and WB MTV > 100 cm^3^ (HR 2.887; *P* = 0.016) showed worse OS, but not PFS.	^279^PMID: 28643145
SPECT	^111^In-DTPA-trastuzumab	Trastuzumab	HER2	Breast cancer	17	Radiolabeled trastuzumab scintigraphy was not valuable in predicting trastuzumab-related cardiotoxicity in metastatic breast cancer patients, but could identify HER2-positive tumors.	^253^PMID: 16710024 (2006)
PET	^89^Zr-trastuzumab	T-dm1	HER2	Breast cancer	55	Among 55 evaluable patients, the negative predictive value (NPV)/positive predictive value (PPV) for T-DM1 response after three cycles were 88%/72% versus 83%/96% for HER2-PET/CT and early FDG-PET/CT separately.	^251^PMID: 26598545 (2016)
PET	^18^F-FDG	Ribociclib, palbociclib or abemcaciclib	CDK4/6	Hormone receptor-positive HER2-negative (HR+/HER2−) metastasized breast cancer	8	Patients with disease control had a significantly greater decline in TLG (−55.3% vs. 16.7%; *P* < 0.05). The same was true for PERCIST-5 (−21.9 vs. 11.3%, *P* < 0.05). All patients with progressive TLG showed treatment failure and/or a poor outcome.	^283^PMID: 34102639
PET	^18^F-FDG	Palbociclib	CDK4/6	Metastatic ER-positive and HER2-(ER+/HER2−) negative BC patients.	12	Compared with Standard Response Evaluation (SRE, based on clinico-laboratory and morphological data), Metabolic Response Evaluation (MRE, based on PET/CT) increased the proportion of patients classified with progressive disease from 25% to 50% and differed from SRE in 8/12 patients: 3/8 shifted from stable disease or undetermined response to metabolic progression (more unfavorable category), 4/8 from stable disease to partial or complete metabolic response, and 1/8 from partial response to complete metabolic response (more favorable category).	^203^PMID: 30569442
PET	^18^F-FES ^18^F-FDG	Letrozole combined with CDK inhibition	ER and CDK4/6	Metastatic ER-positive and HER2-negative BC patients.	30	Median time to progression (TTP) was 73 weeks [95% confidence interval (CI) 21 to ∞] in 7 patients with 100% FES-positive disease, 27 weeks (14–49) in heterogeneous FES-positive disease (20 patients), and 15 weeks (9 to ∞) without FES positivity (three patients; log-rank *P* = 0.30). Geometric mean FES uptake was 2.3 for metabolic progressive patients, 2.5 (*P* vs. progression = 0.82) for metabolic stable disease, and 3.3 (*P* vs. progression = 0.40) for metabolic response (Ptrend = 0.21).	^282^PMID: 31891878
PET	^18^F-FES	Palbociclib combined with endocrine therapy	ER and CDK4/6	ER+/HER2− metastatic breast cancer (MBC) patients	56	Nine out of 10 patients with an 18F-FES-negative site developed PD, and the median PFS was only 2.4 months. Among 46 patients with only 18F-FES-positive lesions, only four patients had PD, and the median PFS was 23.6 months. There were statistically significant differences between the two groups (*P* < 0.001). For the subgroup of patients with only 18F-FES-positive lesions, low FES-HI patients experienced substantially longer PFS times than those with high FES-HI (26.5 months vs. 16.5 months, *P* = 0.004).	^265^PMID: 36028895
PET	^18^F-FDG	Everolimus	mTOR	Metastatic renal cell cancer	63	Relative change in average SUV_max_ was the best predictor of change in tumor burden (all evaluable *P* = 0.01; clear cell subtype *P* = 0.02), with a modest correlation. Baseline average SUV_max_ was correlated with overall survival and progression-free survival (PFS) (*P* = 0.023; 0.020), but not with a change in tumor burden.	^204^PMID: 24156027
PET	^18^F-FDG	Everolimus	mTOR	Advanced neuroendocrine neoplasia	66	Patients in the high (avgSUV_max_ > 4)-uptake group had worse outcomes for both OS (HR = 3.99, *P* = 0.023) and PFS (HR = 2.85, *P* = 0.02).	^284^PMID: 32767279
PET	^89^Zr-atezolizumab	Atezolizumab	PD-L1	Bladder cancer (9) non-small-cell lung cancer (NSCLC) (9) triple-negative breast cancer (TNBC) (4)	22	Clinical responses in patients were better correlated with pretreatment PET signal than with immunohistochemistry- or RNA-sequencing-based predictive biomarkers.	^168^PMID: 30478423 (2018.12)
PET	^18^F-BMS-986192 ^89^Zr-nivolumab	Anti-PD-L1 Adnectin and Nivolumab	PD-L1	NSCLC	13	^18^F-BMS-986192 SUV_peak_ was higher for responding lesions than for nonresponding ones (median 6.5 vs. 3.2, *P* = 0.03), and an analogous lesional correlation was noted for ^89^Zr-nivolumab (median 6.4 vs. 3.9, *P* = 0.019).	^269^PMID: 30405135 (2018.11)
PET	^89^Zr-durvalumab	Durvalumab	PD-L1	NSCLC	13	Tumor uptake was higher in patients with treatment response or stable disease than in patients with disease progression. However, this difference was not statistically significant (median SUV_peak_, 4.9 vs. 2.4; *P* = 0.06).	^267^PMID: 34385342 (2022)
PET	^68^Ga-NOTA-WL12	PD-L1-binding peptide	PD-L1	NSCLC	9	A strong positive correlation was found between tumor uptake and PD-L1 IHC results. Patients with the partial metabolic response (PMR)/stable disease exhibited positivity for uptake of ^68^Ga-NOTA-WL12 before therapy.	^363^PMID:34326125 (2022)
PET	^89^Zr-pembrolizumab	Pembrolizumab	PD-1	Melanoma (11) NSCLC (7)	18	Tumor ^89^Zr-pembrolizumab uptake correlated with tumor response (*P* = 0.014) and progression-free (*P* = 0.0025) and overall survival (*P* = 0.026).	^268^PMID: 34736925 (2022)
PET	^89^Zr-pembrolizumab	Pembrolizumab	PD-1	NSCLC	12	Uptake was higher in patients with a response to pembrolizumab treatment (*n* = 3) than in patients without a response (*n* = 9), although this was not statistically significant (median SUV_peak_, 11.4 vs. 5.7; *P* = 0.066).	^270^PMID:34272316 (2022)

### Evaluation of therapeutic responses

In clinical practice, ^18^F-labeled fluorodeoxyglucose (^18^F-FDG) is the most commonly used radiotracer for PET-based imaging. PET/CT with 18F-FDG has been a proven staging modality for various neoplasms for many years. Besides staging, it is used increasingly frequently to categorize the metabolic response to antineoplastic therapy, called PET Response Criteria in Solid Tumors (PERCIST).^193^ Several studies have confirmed that ^18^F-FDG PET or PET/CT can predict the response to EGFR-TKI treatment in 1–2 weeks, while conventional CT requires 2–3 months in patients with lung cancer.^194–198^ Similar results were also seen upon the use of HER2-targeted therapy,^199^ antiangiogenic agents,^200–202^ CDK4/6 kinase inhibitor,^203^ and mTOR inhibitor.^204^ Therefore, by using ^18^F-FDG, it is possible to evaluate the efficacy of targeted therapy at an early stage without waiting until the middle or end of treatment. For example, in patients with metastatic renal cell cancer treated with an mTOR inhibitor everolimus, the 2-week relative changes of ^18^F-FDG uptake (SUV_max_) from baseline were predictive of the 8-week change in tumor size as evaluated by conventional computed tomography.^204^

^18^F-FLT is another PET tracer for assessing cell proliferation in vivo. It is also a potential candidate for evaluating response to targeted therapy, such as VEGFR TKI sunitinib,^205^ EGFR inhibitors,^206^ HER2 inhibitor trastuzumab,^207^ PI3K inhibitor GDC-0941, and the MEK inhibitor PD 0325901,^208^ mTOR inhibition everolimus,^209^ but most of the studies were in preclinical models.

Despite the optimal performance of ^18^F-FDG or ^18^F-FLT in a clinical context, they suffer from certain limitations in evaluating therapeutic response as it only detects the glucose metabolism of tumors and cannot reflect all biometric features of tumors. Against this background, many studies are underway to explore and develop specific radiotracers capable of binding to particular targets and to identify efficacy as accurately as possible.

In addition to ^18^F, some other PET radioisotopes are commonly used, including ^124^I, ^89^Zr, ^68^Ga, and ^64^Cu.^210^ Among these, ^124^I and ^89^Zr with long half-lives have been used for radiolabeling intact antibodies. In contrast, short half-life nuclides have been used for antibody fragments, nanobodies, peptides, affibodies, and small molecules.^211^

For antiangiogenic therapy, iodinated VEGF is the most studied radiolabeled VEGF tracer.^212–215^ VEGF, VEGFR, and related integrins can also be labeled with ^18^F,^216,217^ ^99^mTc,^218–220^ ^111^In,^220,221^ ^64^Cu,^218,222,223^ and ^89^Zr,^224^ but most of these are simply for imaging of the tumor vasculature and can help to visualize tumors and metastatic lesions usually overexpressing VEGFR or VEGF. Recently, reports have been published on several clinical trials on the efficacy of ^89^Zr-labeled bevacizumab imaging for monitoring various cancer-targeted therapy such as the mTOR inhibitor everolimus,^225^ bevacizumab/interferon-α,^226^ multi-targeted tyrosine kinase inhibitors like sunitinib,^226^ and a similar result was found in ^111^In-bevacizumab imaging for evaluation sorafenib.^111^ In 70 evaluable lesions of 10 patients with metastatic renal cell carcinoma, the uptake of ^89^Zr-bevacizumab (SUV_max_) decreased by a mean of 9.1% (*P* < 0.0001) at 2 weeks and 23.4% (*P* < 0.0001) at 6 weeks after everolimus treatment. All 10 patients continued the treatment and had stable disease at 3 months.^225^ Similar data were found in patients with metastatic renal cell carcinomas treated with bevacizumab/interferon-α.^226^ In patients who received sunitinib from the same study, the uptake of ^89^Zr-bevacizumab (SUV_max_) decreased by 14.3% (*P* = 0.006) at 2 weeks but increased by 72.6% (*P* < 0.0001) at 6 weeks after treatment compared with that at baseline.^226^ These data indicate the role of ^89^Zr-bevacizumab imaging in reflecting the biological effects of cancer-targeted therapy.

Evaluation of the success of HER2-targeted therapy depends on the precise determination of HER2 expression. Full-length monoclonal antibodies are usually labeled with long-half-life radionuclides, such as ^64^Cu,^227–231^ ^89^Zr,^232–234^ ^111^In,^235,236^ and ^124^I.^237^ Besides, imaging tracers targeting HER2 by shorter fragments (antibody fragments [Fab or F(ab)2], nanobodies, or affibodies) have been introduced into clinical trials, such as ^68^Ga-DOTA-F(ab0)2-trastuzumab,^238^ ^68^Ga-NOTA-2Rs15d,^171^ ABY-002 labeled with ^111^In and ^68^Ga,^239^ ^111^In-ABY-025,^174^ ^68^Ga-ABY-025,^175^ ^99^mTc-ADAPT6,^240^ and ^99^mTc-(HE)3-G3,^241^ to assess HER2 expression in breast carcinoma.

Most anti-HER2 probes exhibit the potential advantages of enabling the noninvasive and specific identification of HER2-positive tumors. In a study by Guo et al. ^242^, PET imaging of ^124^I-trastuzumab showed a difference in SUV_max_ (7.83 ± 0.55 vs. 1.75 ± 0.29, *P* < 0.0001) between HER2-positive and -negative lesions and recognized 18 out of 18 HER2-positive lesions in both primary and metastatic gastric cancer patients. In addition, Ulaner and colleagues published a series of articles about ^89^Zr-trastuzumab^243,244^ and ^89^Zr-pertuzumab^245,246^ to identify HER2-positive metastases in patients with HER2-negative primary breast cancer. Similar results were confirmed for ^64^Cu-DOTA-trastuzumab^228,247,248^ in breast cancer. Besides screening HER2-positive lesions, some novel molecular imaging probes were designed to monitor the response to anti-HER2 treatment directly.^249–251^ In a preclinical study, ^89^Zr-pertuzumab accurately detected changes in tumor volume from 243.80 ± 40.91 mm^3^ before T-DM1 therapy to 78.4 ± 40.43 mm^3^ after this therapy in mice bearing BT-474 tumors. In contrast to the findings with ^89^Zr-pertuzumab, no apparent changes were observed in ^18^F-FDG.^249^ Similar preclinical results were obtained with ^99^mTc-HYNIC-H10F, which can assess trastuzumab response at the earlier stage of day 4 post-treatment.^250^ In addition, in 2021, a study reported that ^64^Cu-DOTA-trastuzumab could predict the response of metastatic breast cancer patients receiving T-DM1.^252^ When compared with those in nonresponding patients, higher minimum SUV_max_ (5.6 vs. 2.8, *P* < 0.02) at day 1, higher average SUV_max_ (8.5 vs. 5.4, *P* < 0.05), and higher minimum SUV_max_ (8.1 vs. 3.2, *P* < 0.01) at day 2 were found in responding patients.

For trastuzumab-related cardiotoxicity, Perik et al. found that myocardial ^111^In-DTPA-trastuzumab uptake was not associated with cardiotoxicity in all 15 evaluable HER2-positive metastatic breast cancer patients.^253^ Next, they discovered that myocardial ^111^In-DTPA-trastuzumab uptake was observed in 50% of anthracycline-treated patients without symptomatic cardiac dysfunction, while none was found in non-anthracycline-related heart failure patients. They thus considered that ^111^In-DTPA-trastuzumab potentially recognized patients susceptible to trastuzumab-related cardiotoxicity.^254^

For EGFR-targeted therapy, cetuximab, panitumumab, and their analogs were labeled with different radionuclides. Most of the tracers, such as ^18^F-FBEM-cEGF (ligand),^255^ ^64^Cu-panitumumab,^256^ ^111^In-cetuximab,^257,258^ ^64^Cu-cetuximab,^258^ ^89^Zr-cetuximab,^259^ ^111^In-cetuximab-F(ab’)2 (antibody fragment),^260^ ^64^Cu-cetuximab-F(ab’)2 (antibody fragment),^261^ and ^89^Zr-DFO-ZEGFR:2377 (affibody),^262^ were designed to image the expression of EGFR in vivo. In 10 advanced colorectal cancer (mCRC) patients without K-RAS mutation, ^89^Zr-cetuximab uptake was found in 6 patients, 4 of whom benefited from cetuximab treatment. Disease progression was detected in 3 of the remaining 4 patients without uptake of ^89^Zr-cetuximab. The results suggested that ^89^Zr-cetuximab uptake is correlated with response, but this warrants further clinical validation.^263^ Besides, N-(3-chloro-4-fluorophenyl)-7-(2-(2-(2-(2-18F-fluoroethoxy) ethoxy) ethoxy) ethoxy)-6-methoxyquinazolin-4-amine (^18^F-MPG) precisely quantified EGFR-activating mutation status, meanwhile monitored the response to EGFR-TKIs in NSCLC patients.^264^

To predict the treatment response of CDK4/6 inhibitors combined with endocrine therapy in ER+/HER2-metastatic breast cancer (MBC) patients, ^18^F-FES-PET/CT was undergone. 90% ^18^F-FES-negative patients developed progressive disease (PD), while only 8.7% ^18^F-FES-positive patients had PD. ^18^F-FES-positive patients had longer PFS than ^18^F-FES-negative patients (23.6 months vs. 2.4 months, *P* < 0.001).^265^

**Fig. 4:**
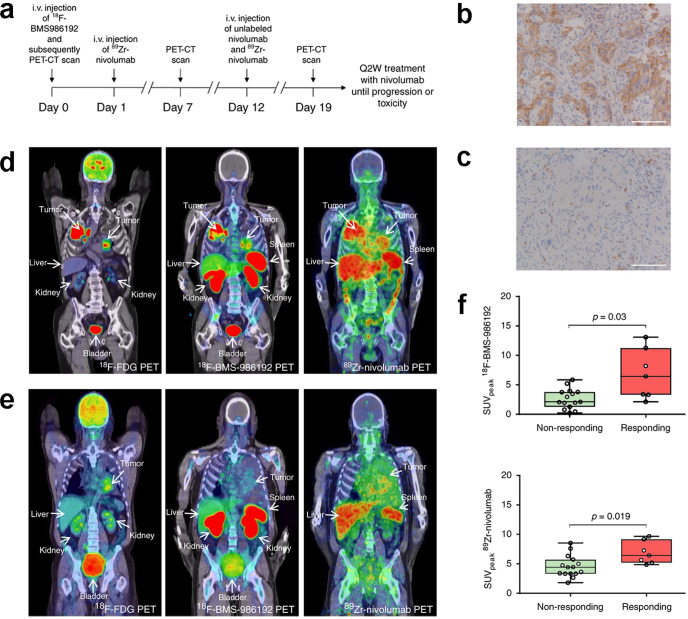
Tracer uptake and correlation with PD1/PDL1 treatment response. **a** Study design. **b** Immunohistochemical staining of PD-L1 in patient 2. Biopsy of the tumor in the left lower lobe. PD-L1 expression is expressed in 95% of the tumor cells. Scale bar, 100 µm. **c** Immunohistochemical staining of PD-1 in patient 2. PD-1 expression in aggregates was scored as IC1. Scale bar, 100 µm. **d** ^18^F-FDG PET (225 MBq) (^18^F-FDG PET scan images of both patients were used from archival PET scans) demonstrates high glucose metabolism of tumors in both lungs and mediastinal lymph nodes. ^18^F-BMS-986192 PET (145.7 MBq, imaging time point 1 h post-injection (p.i.)) and ^89^Zr-labeled Nivolumab PET (37.09 MBq, 162 h p.i.) demonstrate heterogeneous tracer uptake within and between tumors. **e** Patient 3 with tumor PD-L1 expression < 1%: ^18^F-FDG PET (268 MBq) (18F-FDG PET scan images of both patients were used from archival PET scans) demonstrates high glucose metabolism in the left-sided tumor. ^18^F -BMS-986192 PET (214.62 MBq, 1 h p.i.) demonstrates low tumor tracer uptake. ^89^Zr-labeled Nivolumab PET (37.27 MBq, 160 h p.i.) demonstrates heterogeneous tracer uptake in the tumor. **f** SUV_peak_ of the ^18^F-BMS-986192 tracer is higher in responding lesions than non-responding lesions (comparison of lesions with a diameter of 20 mm or more). The *p*-value is 0.02, as determined by the Mann–Whitney *U*-test. SUV_peak_ of the ^89^Zr-nivolumab tracer is numerically higher in responding lesions (comparison of lesions with a diameter of 20 mm or more). The *p*-value is 0.019, as determined by the Mann–Whitney *U*-test. For all the boxplots, the lower edge of the box represents the first quartile, and the upper edge represents the third quartile. The horizontal line inside the box indicates the median. Whiskers identify the minimum and the maximum value. (Reproduced from Niemeijer, A. N. et al. Whole body PD-1 and PD-L1 positron emission tomography in patients with non-small-cell lung cancer. *Nat. Commun.* **9**, 4664 (2018))

Immunotherapy is associated with pseudo-progression, which limits the application of conventional anatomically based imaging modalities for treatment evaluation. Recently, some studies have demonstrated the use of radiolabeled PD-L1 antibodies (^68^Ga-NOTA-Nb10989, ^89^Zr-atezolizumab, ^89^Zr-durvalumab)^168,266,267^ and PD-1 antibodies (^89^Zr-pembrolizumab, ^89^Zr-nivolumab)^268–270^ to assess the efficacy of PD-1/PD-L1 blockade in cancer patients. The first-in-human whole-body PD-L1 imaging study by Niemeijer et al. used ^18^F-BMS-986192 and ^89^Zr-nivolumab in 13 NSCLC patients with nivolumab treatment^269^(see Fig. 4). They showed that median SUV_peak_ values of ^18^F-BMS-986192 (6.5 vs. 3.2, *P* = 0.03) and ^89^Zr-nivolumab (6.4 vs. 3.9, *P* = 0.019) were higher in responding lesions than in nonresponding ones. Similar results were obtained for using ^89^Zr-atezolizumab to assess atezolizumab response in 22 patients, including 9 bladder cancer patients, 9 NSCLC patients, and 4 triple-negative breast cancer patients.^168^

Therefore, ^18^F-FDG PET is potentially useful for the early evaluation of therapeutic response. Tumor-specific nuclear imaging holds promise to assess the target expression and to predict the targeted treatment response early.

### Prognosis prediction

Radionuclide imaging can also predict the prognosis of patients prescribed targeted therapy. As the most widely used tracer in oncology, ^18^F-FDG has been investigated in the early prediction of outcomes after targeted treatment, including that with anti-EGFR agents such as the monoclonal antibody cetuximab.^271^ It was reported that an increase in peak tumor metabolism at the end of the first week of therapy implied poor PFS (*P* = 0.001) and OS (*P* < 0.001) at the end of the first week of third-line cetuximab-based therapy in metastatic colorectal cancer patients. Similar results were also found for the EGFR-TKIs erlotinib and gefitinib,^272–275^ the antiangiogenic agent bevacizumab,^202,276–278^ HER2-targeted therapy with trastuzumab,^279^ multiple TKIs such as regorafenib,^280,281^ the CDK4/6 kinase inhibitors ribociclib, palbociclib, and abemcaciclib,^203,282,283^ and the mTOR inhibitor everolimus,^204,284^ as well as the ICIs nivolumab and pembrolizumab.^285,286^ In a meta-analysis,^287^ 865 participants from 26 studies receiving inspection of FDG-PET or FLT-PET were included. In comparison with the PET nonresponsive group, the PET responsive group showed a decline in SUV_max_, which was related to prolonged PFS (HR = 0.41, *P* < 0.00001), OS (HR = 0.52, *P* < 0.00001), and time to progression (TTP) (HR = 0.30, *P* = 0.003). However, some other clinical trials obtained inconsistent results on this issue. Rinzivillo et al. found no significant difference in clinical outcomes between ^18^F-FDG-PET-positive and -negative groups in patients with advanced neuroendocrine neoplasia receiving everolimus therapy (median PFS of 24 and 18 months, respectively; *P* = 0.337).^284^ In general, the prognostic value of ^18^F-FDG-PET in patients who received targeted therapy is inconclusive. The prediction based on ^18^F-FDG-PET imaging may be cancer-specific or treatment-specific.

To clarify the accuracy of PET or SPECT for predicting prognosis, especially in patients who have simultaneously received two or more combined targeted therapies or ICIs, a few specific radiotracers are under pre-clinical and clinical investigations. Rainer et al. investigated the predictive value of ^123^I-VEGF165 scintigraphy in patients with glioma.^213^ Their results showed that ^123^I-VEGF165 may provide relevant prognostic information in glioma, as patients with a tumor-to-normal brain uptake ratio (T/N ratio) of <1.32 showed significantly longer survival (2680 days vs. 295 days; *P* < 0.05). Similar results were seen in a grade IV glioma subgroup, as patients with a T/N ratio < 1.75 had longer OS (720 vs. 183 days; *P* < 0.05).^288^ ^89^Zr-bevacizumab^226^ was used to predict differential effects of antiangiogenic treatment (bevacizumab with interferon-α or sunitinib) in metastatic RCC (mRCC). High baseline tumor SUV_max_ before antiangiogenic therapy in the three most intense lesions was correlated with longer time to disease progression (89.7 vs. 23.0 weeks; HR = 0.22; *P* = 0.050).

For HER2-targeted therapy, in 2016, Gebhart et al. ^251^ conducted a prospective clinical trial (ZEPHIR study) using ^89^Zr-trastuzumab PET/CT and FDG PET/CT to predict the efficacy of T-DMl therapy in patients with HER2-positive metastatic breast cancer. The results showed that, among the 55 evaluable patients, the negative predictive value was 100% when combining ^89^Zr-trastuzumab-PET with early FDG-PET response after one cycle of T-DM1, which can predict response to T-DM1 and differentiate patients with a median time to treatment failure (TTF) of 2.8 months from those with a TTF of 15 months.

For EGFR-targeted therapy, even though FDG-PET/CT is better than CT at assessing the benefit of cetuximab in incurable squamous cell carcinoma of the head and neck,^289^ no relationship was identified between uptake on ^89^Zr-cetuximab PET/CT and PFS (3.6 vs. 5.7 months, *P* = 0.15) or OS (7.1 vs. 9.4 months, *P* = 0.29) in patients with RAS wild-type advanced colorectal cancer receiving cetuximab monotherapy.

For immunotherapy, it was reported that high tumor uptake of ^89^Zr-atezolizumab was correlated with a better response to atezolizumab treatment. Those with low uptake were more likely to progress or die, whereas PD-L1 IHC failed to predict the treatment outcome.^168^ Similar results were seen for ^89^Zr-pembrolizumab.^268^

Taking together, immuno-PET imaging, as a non-invasive method for the early detection of tumor receptor blocking by anti-cancer targeted drugs, may serve as an effective technique in predicting patient prognosis.

### MRI imaging

Regarding the clinical application of MRI in cancer-targeted therapy, the focus has been particularly placed on three aspects: (1) predicting the response to targeted therapy by using information obtained by MRI performed pretreatment; (2) evaluating the treatment response to targeted therapy by analyzing the changes in MRI parameters; and (3) predicting patient outcome in those receiving targeted therapy. Data on the clinical application of MRI in cancer-targeted therapy are summarized in Table 3.

**Table 3:** Clinical application of MRI in cancer-targeted therapy

Technique	Drug	Target	Tumor	*N*	Results	References
DCE-MRI	Bevacizumab	VEGF	Breast cancer	70	Significant decreases in *K*^trans^, *k*_ep_, *V*_e_, and AUC_60_ after cycle 5 of treatment.	^291^PMID: 34298725
DCE-MRI	Bevacizumab (+chemotherapy)	VEGF	Breast cancer	19	Significant decreases in *K*^trans^, *k*_ep_, and IAUGC at 180 s after cycle 1 of treatment. The median relative change in the slope of the wash-in curve from baseline to cycle 4 was significantly different between responders and nonresponders.	^364^PMID: 17709827
DCE-MRI	Bevacizumab (+chemotherapy)	VEGF	Breast cancer	21	Decreases in *K*^trans^, *k*_ep_, and *V*_e_ after cycle 1 of treatment. No correlation with treatment response.	^290^PMID: 16391297
DSC-MRI	Bevacizumab (+radiotherapy)	VEGF	Glioblastoma	67	OS benefit from bevacizumab plus radiotherapy compared with radiotherapy alone was observed for larger baseline MRI contrast-enhancing tumors and for higher ADC.	^300^PMID: 32967939
DSC-MRI	Bevacizumab (+chemotherapy)	VEGF	Glioblastoma	123	Quantitative DT1 showed a significant difference in OS at week 8 between responders and nonresponders/nonprogressors.	^365^PMID: 31248863
DSC-MRI	Bevacizumab (+chemotherapy)	VEGF	Glioblastoma	254	Decreases in nrCBV, nrCBF, and nTMRO_2_ values after bevacizumab treatment. None of these parameters was predictive of OS.	^302^PMID: 32720870
DSC-MRI	Bevacizumab (+chemotherapy)	VEGF	Glioblastoma	21	Early decreases in rCBV were predictive of improved survival.	^303^PMID: 25646027
DSC-MRI and DCE-MRI	Bevacizumab (+chemoradiation therapy)	VEGF	Glioblastoma	42	High pretreatment rCBV was predictive of improved OS.	^305^PMID: 32678438
DSC-MRI and DCE-MRI	Bevacizumab (+chemotherapy)	VEGF	Breast cancer	22	A lower Δ*K*^trans^ or ΔADC reduction in 21 days after treatment predicted shorter CNS-specific PFS. A lower ΔPeak or ΔIAUC_60_ reduction predicted shorter OS.	^295^PMID: 29770848
DSC-MRI and DCE-MRI	Bevacizumab (+chemotherapy)	VEGF	Glioblastoma	33	PFS increased significantly with time to the maximum value of the residue (*T*_max_). OS decreased significantly with srCBV and increased significantly with *T*_max_.	^366^PMID: 33828310
MRS	Bevacizumab (+chemotherapy)	VEGF	Glioblastoma	13	Increased NAA/Cho at 8 weeks and decreased Cho/Cr and increased NAA/Cr and NAA/Cho at 16 weeks post-treatment was associated with both 6-month progression-free survival and 1-year survival.	^317^PMID: 23645534
MRS	Bevacizumab (+chemotherapy)	VEGF	Glioblastoma	21	A lower mI/c-Cr in the intratumoral and peritumoral volume before and during treatment was predictive of poor survival.	^367^PMID: 34751617
VHL and VAM	Bevacizumab	VEGF	Glioblastoma	13	Early response to bevacizumab was dominated by the reduction of smaller microvasculature.	^326^PMID: 28819189
DWI	Bevacizumab (+chemotherapy)	VEGF	Glioblastoma	123	High pretreatment contrast-enhancing tumor volume was associated with shorter PFS and OS. A high volume fraction of increasing ADC after therapy was associated with shorter PFS, while a high volume fraction of decreasing ADC was associated with shorter OS.	^301^PMID: 25672376
DWI	Bevacizumab (+chemotherapy)	VEGF	Colorectal liver metastasis	74	Post-treatment ADC_mean_ was significantly associated with OS and PFS.	^312^PMID: 35013857
DWI	Bevacizumab (+chemotherapy)	VEGF	Glioblastoma	32	Pretreatment tumor volume was correlated with OS. Patients with high ADCL had favorable survival when treated with bevacizumab.	^313^PMID: 32365185
DWI	Bevacizumab (+chemotherapy)	VEGF	Glioblastoma	242	ADC_low_ was an independent prognostic parameter for OS and PFS. Patients with ADC_low_ ≥ 1241 × 10^−6^ mm²/s had prolonged OS compared with those with ADC_low_ < 1241 × 10^−6^ mm²/s.	^314^PMID: 32393964
DCE-MRI and DWI	Bevacizumab (+chemotherapy)	VEGF	Colorectal liver metastasis	126	D-RECIST- but not RECIST-defined responders had significantly longer median DFS than nonresponders. D-RECIST- but not RECIST-defined responses independently predicted DFS.	^310^PMID: 33449175
APT MRI and DWI	Bevacizumab	VEGF	Glioblastoma	54	Mean APT signal intensity change after bevacizumab treatment indicated a low 12-month progression rate and longer PFS. High mean normalized CBV at follow-up was associated with a high 12-month progression rate and shorter PFS. Mean APT signal intensity change was a significant predictor of diffuse non-enhancing progression, whereas follow-up 95th percentile of the normalized CBV was a predictor of local enhancing progression.	^323^PMID: 32154775
CEST- EPI	Bevacizumab (with or without adjuvant chemotherapy or immunotherapy)	VEGF	Glioblastoma	11	The reduction in tumor acidity was linearly correlated with PFS, being a significant predictor of PFS.	^322^PMID: 30806888
DCE-MRI and FLAIR	Bevacizumab (+chemoradiation therapy)	VEGF	Glioblastoma	159	Increasing 2D-T1 and FLAIR post-treatment significantly predicted worse OS. Adjusting for 2D-T1 and treatment, increasing FLAIR represented a significantly higher risk for death.	^368^PMID: 29590461
FLAIR	Bevacizumab (with or without chemotherapy)	VEGF	Gliomas	33	Lower edge contrast of the FLAIR hyperintense region was associated with poorer PFS and OS.	^369^PMID: 29622553
DCE-MRI and FLAIR	Bevacizumab (with or without chemotherapy)	VEGF	Glioblastoma	119	Early MRI response could predict PFS and OS. Early MRI progression was a strong independent predictor of mortality.	^370^PMID: 28678383
T2WI and DCE-MRI	Bevacizumab (+chemoradiation therapy)	VEGF	Glioblastoma	232	At weeks 6 and 12 of treatment, increases in baseline necrosis and de novo necrosis were strongly associated with worse OS and PFS.	^371^PMID: 31076534
TME mapping	Bevacizumab	VEGF	Glioblastoma	18	Higher percentage of neovascularization and active tumor in baseline indicated poor or no treatment response.	^329^PMID: 30361791
PWI	Angiocept, bevacizumab, cilengitide, enzastaurin, sorafenib, thalidomide and vandetani	VEGF	Glioblastoma	117	Patients with an angiogenic subtype of glioblastoma benefited from antiangiogenic therapy with improved OS.	^372^PMID: 28007759
DCE-MRI and DWI	Bevacizumab or aflibercept or cediranib or cabozantinib	VEGF	Glioblastoma	258	Baseline ADC_L_ was an independent predictive biomarker for OS in anti-VEGF therapies. An ADC_L_ threshold of 1.24 μm^2^/ms produced the largest OS differences between patients.	^309^PMID: 28655794
DWI-MRI	Lenvatinib and toripalimab	VEGF	Intrahepatic cholangiocarcinomas	43	ADC was an independent variable associated with early progression. Patients with low ADC values showed shorter PFS.	^307^PMID: 35488518
Gd-EOB-DTPA-enhanced MRI	Lenvatinib or atezolizumab and bevacizumab	VEGF	Hepatocellular carcinoma	68	No predictive association between PFS and EOB-MRI in the lenvatinib group. In the atezolizumab plus bevacizumab group, the heterogeneous type and hyperintensity type had significantly shorter PFS than the homogeneous type and the hypointensity type, respectively.	^332^PMID: 35159095
DSC-MRI and DCE-MRI	Cabozantinib	VEGF	Glioblastoma	108	A log-linear association between baseline tumor volume and OS and a linear correlation between initial change in tumor volume and OS were observed. Continuous measures of baseline tumor volume and volumetric response were independent predictors of OS.	^299^PMID: 29660005
DSC-MRI and DCE-MRI	Lenalidomide or axitinib	VEGF	Hepatocellular carcinoma	74	Greater reductions in ΔPeak or ΔAUC on days 3 and 14, and Δ*K*^trans^ on day 14 were associated with better PFS. Greater reductions in ΔAUC or Δ*K*^trans^ on day 14 were associated with better OS. Δ*K*^trans^ on day 14 was an independent predictor of PFS after controlling for ORR and DCR.	^296^PMID: 34638446
ASL MRI	Sunitinib or pazopanib	VEGF	Renal cell carcinoma	28	Responders had higher baseline tumor perfusion than nonresponders. Interval reductions in perfusion at week 2, cycle 2, and cycle 4 were not associated with ORR or PFS.	^327^PMID: 33258745
DWI	Sunitinib, pazopanib or axitinib	VEGF	Renal cell carcinoma	92	Patients with >5 bone metastases (BM) on WB-DWI/MRI had a lower response rate, and more frequently suffered early progressive disease, shorter PFS, and shorter OS than patients with ≤5 BM.	^373^PMID: 32297532
DWI	Sunitinib	VEGF	Gastrointestinal stromal tumor	15	Pretreatment β and ΔD differed between good- and poor-responding lesions. Combining Δ*D* with pretreatment β obtained an improved AUC (0.843) with a predictive accuracy of 75.7%.	^308^PMID: 28643387
DCE-MRI and FLAIR	Sunitinib	VEGF	Renal cell carcinoma	34	Higher baseline and day 14 values for K^trans^ were significantly associated with longer PFS.	^374^PMID: 29383520
DWI	Imatinib or sunitinib	VEGF	Gastrointestinal stromal tumor	62	The percentage change of ADC and longest diameter after 2 weeks of therapy were significantly different between responders and nonresponders.	^306^PMID: 30103713
DCE-MRI	Regorafenib	VEGF	Colorectal cancer	27	>70% drop in KEF (6/23) was associated with a higher disease control rate at 2 months and improved PFS and OS.	^297^PMID: 28790159
DCE-MRI	Sorafenib	VEGF	Hepatocellular carcinoma	29	Stratification according to mRECIST and vqEASL successfully captured response and stratified OS, while stratification according to RECIST and %qEASL did not correlate with OS.	^298^PMID: 33123796
Gd-EOB-DTPA-enhanced MRI	Sorafenib (with or without selective internal radiation therapy)	VEGF	Hepatocellular carcinoma	312	High gadoxetic acid uptake on pretreatment MRI was significantly associated with shorter OS.	^375^PMID: 34541612
Gd-EOB-DTPA-enhanced MRI	Sorafenib (with or without selective internal radiation therapy)	VEGF	Hepatocellular carcinoma	376	Peritumoral arterial enhancement and peritumoral hypointensity in hepatobiliary phase were predictors of worse OS. Peritumoral hypointensity in hepatobiliary phase was a predictor of liver decompensation.	^376^PMID: 34686780
Gd-EOB-DTPA-enhanced MRI	Sorafenib	VEGF	Hepatocellular carcinoma	65	Regular tumor margin and the presence of tumor thrombus were indicators of high RAF1 expression.	^377^PMID: 34738148
Gd-EOB-DTPA-enhanced MRI	Sorafenib	VEGF	Hepatocellular carcinoma	91	The presence of incomplete capsule or intratumoral vessels and the absence of capsule were potential indicators of high BRAF and RAF1 expression.	^378^PMID: 30547202
MRE	Sorafenib	VEGF	Hepatocellular carcinoma	50	Higher MRE-assessed liver stiffness was significantly associated with poor OS and significant liver injury after sorafenib therapy.	^328^PMID: 33033862
MRS	Cediranib (+chemoradiation therapy)	VEFG	Glioblastoma	40	Total Cho/healthy Cr after 1 month of treatment was significantly associated with OS.	^379^PMID: 29202103
IVIM-MRI and DCE-MRI	Lenalidomide	VEGF	Hepatocellular carcinoma	44	Participants with a higher slope, *K*_ep_ and ADC values had longer PFS. Participants with small tumor size, higher slope, ADC and *f* values had longer OS. *K*_ep_ and ADC were independent predictors of PFS. Slope and ADC were independent predictors of OS.	^325^PMID: 34441274
DCE-MRI	Bevacizumab and erlotinib	VEGF and EGFR	NSCLC	44	Whole-tumor *K*^trans^ was not associated with PFS, but patients with an increase of more than 15% in the SD of tumor *K*^trans^ values after 3 weeks had shorter PFS.	^200^PMID: 21149474
DWI	Bevacizumab and erlotinib (+chemoradiation therapy)	VEGF and EGFR	Glioblastoma	36	A lower ADC percentile value within the T2-hyperintense lesion (T2L) at early follow-up timepoints was associated with worse outcomes. The ADC10% within the T2L at 2 months was strongly associated with OS and PFS.	^315^PMID: 25351579
DCE-MRI and DWI	Gefitinib (+radiotherapy)	EGFR	Nonsmall-cell lung cancer	253	Tumor regression rate, ADC_post_, ΔADC_post_, and ΔADC_post_ (%) were key imaging indicators for predicting the outcome.	^311^PMID: 34514171
DCE-MRI	Trastuzumab or T-DM1 ( + chemotherapy)	HER2	Breast cancer	46	Interim changes in ETV value were highly correlated with residual cancer burden.	^380^PMID: 29641224
DCE-MRI	Trastuzumab or/and pertuzumab	HER2	Breast cancer	21	Concentric tumor shrinkage pattern after targeted therapy was associated with pCR. No association between the initial enhancement ratio and pCR.	^294^PMID: 31444111
DSC-MRI	Trastuzumab (+chemotherapy)	HER2	Breast cancer	296	Patients with early rCR on MRI achieved pCR in 73% of HER2-positive breast cancer cases and 88% in the HR-negative subgroup. Achieving rCR was associated with a rate of the 5-year recurrence-free interval of 88%, compared with 68% without rCR.	^381^PMID: 28432515
IVIM-MRI	Nivolumab or pembrolizumab	PD-1	Non-small cell lung cancer (NSCLC)	20	An increased ADC at 8 weeks and decreased ADC_kurt_ and ΔADC_kurt_ 4 weeks after treatment were associated with objective responses and longer PFS. A decreased ΔADC_skew_ at 4 weeks was associated with objective responses, disease control, and longer PFS and OS.	^324^PMID: 32203770
Gd-EOB-DTPA-enhanced MRI	Anti-PD-1/PD-L1 monotherapy	PD-1/PD-L1	Hepatocellular carcinoma (HCC)	18	The TTnP and median PFS in HCC patients with hyperintense nodules were significantly shorter than in those with hypointense HCC nodules after treatment.	^331^PMID: 34950184

### DCE-MRI and DSC-MRI

Among the different MRI techniques, DCE-MRI is the approach most commonly studied in evaluating treatment response to targeted therapies. In an early study of 21 patients with inflammatory and locally advanced breast cancer treated with bevacizumab, the DCE-MRI parameters *K*^trans^, *V*_e_, and *K*_ep_ significantly decreased compared with those at baseline after one cycle of bevacizumab.^290^ This was accompanied by a decrease in the tumor expression of p-VEGFR2 and an increase in tumor apoptosis, as evaluated by the TUNEL assay.^290^ Similar reductions in *K*^trans^, *V*_e_, and *K*_ep_ from baseline were observed in 70 patients with early breast cancer who received one cycle of bevacizumab as neoadjuvant therapy.^291^ Besides, the changes in DCE-MRI parameters were significantly correlated to the changes in the SUV of FLT-PET imaging.^291^ In patients with NSCLC under gefitinib or erlotinib therapy, *K*^trans^, *V*_e_, and *V*_p_ decreased significantly at day 7 post-treatment.^292^ These results indicate that the changes in DCE-MRI parameters reflect the biological effects of bevacizumab or EGFR tyrosine kinase inhibitors on tumor cells and may serve as early noninvasive imaging biomarkers for evaluating the response to VEGFR-targeted agents.

Regarding anti-HER2 therapy, in a preclinical study, Syed and coworkers found that, in a mouse model with a HER2-positive tumor, both vascular heterogeneity and cellularity heterogeneity increased after trastuzumab treatment, as indicated by increases in the mean K–S distance for the *K*^trans^ distribution and *V*_e_ distribution, respectively.^293^ An increase in cellularity heterogeneity in trastuzumab-treated tumors is expected with increased tumor cell death. Therefore, the changes in these MRI parameters could potentially be used for evaluating the treatment efficacy of trastuzumab. In a study with 51 HER2-positive breast cancer patients who received neoadjuvant HER2-targeted therapy, the pretreatment initial enhancement ratio, defined as the percentage signal increase relative to the baseline at the first postcontrast acquisition, was not associated with tumor pathological complete response after treatment.^294^ Therefore, the changes in the DCE-MRI parameters of tumors after treatment may better reflect the impact of trastuzumab on tumors and serve as a more helpful tool for evaluating treatment efficacy.

The data from DCE-MRI imaging have also been shown to correlate with patient prognosis. For example, in a study of 22 patients with breast cancer that had metastasized to the brain, small reductions in *K*^trans^ and Peak in DCE-MRI scanning 3 weeks after bevacizumab therapy were independently correlated with shorter central nervous system-specific progression-free survival (PFS) and shorter overall survival (OS), respectively.^295^ Similar results were observed in patients with advanced hepatocellular carcinoma. Significant early reductions in *K*^trans^, Peak, and AUC in the tumor were associated with longer PFS and OS than in those with smaller reductions.^296^ In addition, in patients with metastatic colorectal cancer, a drop in KEF, derived from *K*^trans^ and enhancing fraction (EF) (*K*^trans^ × EF), 15 days after regorafenib treatment was correlated with reduced CD31 expression (a marker of vascular density) in the tumor tissue, indicating the biological effect of the treatment.^297^ Moreover, patients with a >70% reduction in KEF had a higher disease control rate and longer PFS and OS than the remaining patients.^297^ The post-treatment contrast-enhancing volumetric change has been demonstrated to be prognostic. In patients with advanced-stage hepatocellular carcinoma treated with sorafenib, the difference in the enhancing lesion volume after treatment could discriminate patients with tumor progression from those with tumor control. Furthermore, this patient classification was proven to predict the prognosis as defined by OS independently.^298^ The similar predictive value of post-treatment enhancing volumetric change was confirmed in patients with recurrent glioblastoma receiving cabozantinib.^299^ The pretreatment contrast-enhancing volume could also be predictive. In post hoc analysis of the randomized ARTE trial, in which patients newly diagnosed with glioblastoma were treated with radiotherapy with or without bevacizumab, larger pretreatment contrast-enhancing volume was associated with inferior OS in both treatment arms.^300^ This result was supported by data from the ACRIN 6677/RTOG 0625 trial and the EORTC 26101 trial, which included patients with recurrent glioblastoma treated with bevacizumab and chemotherapy.^301,302^ In addition, in both of these trials, early decreases in the relative cerebral blood volume (rCBV), derived from DSC-MRI, after treatment was associated with improved OS.^302,303^ Moreover, in pediatric patients with supratentorial high-grade glioma treated with radiotherapy plus erlotinib, the CBV ratio of tumor to normal brain tissue remained relatively constant after treatment.^304^ Nevertheless, patients with a CBV ratio above 1.15 at 8 weeks after treatment had a shorter time to death than the other patients.^304^ Another study evaluated the predictive value of baseline rCBV before bevacizumab treatment in the ACRIN 6686 trial. Patients with newly diagnosed glioblastoma who had high pretreatment rCBV demonstrated improved OS in the bevacizumab-treated group compared with that in the placebo group.^305^ These findings indicate that monitoring the change in the blood volume of tumors may be more critical than merely learning about their absolute value in evaluating their prognostic significance. Contradictory data regarding the prognostic value of *K*^trans^ and rCBV have been reported in the literature, with the changes in these variables being reported not to be predictive for OS in patients newly diagnosed with glioblastoma.^305^ Moreover, in patients with NSCLC who were treated with bevacizumab and erlotinib, *K*^trans^ was not associated with PFS. However, an increase in *K*^trans^ heterogeneity 3 weeks after treatment was found to be associated with worse PFS.^200^ This discrepancy may have been due to the small number of patients in most of the studies and the different approaches used for defining the changes in *K*^trans^ among the studies.^295,305^

### Diffusion-weighted imaging (DWI)

DWI is another commonly used MRI technique for evaluating the response to anticancer treatments. Tang and colleagues demonstrated that in patients with gastrointestinal stromal tumor (GIST) treated by neoadjuvant imatinib or sunitinib, the percentage change in ADC 2 weeks after therapy differed significantly between responders and nonresponders (increase by 30% in responders vs. an increase by 1% in nonresponders, *P* < 0.001).^306^ Additionally, the predictive value of pretreatment ADC was confirmed in patients with unresectable intrahepatic cholangiocarcinoma (ICC) who received first-line systemic therapy with lenvatinib plus PD1 antibody.^307^ Another DWI-related parameter, the pretreatment fractional order parameter β (which correlates with intravoxel tissue heterogeneity), was proven to be predictive of treatment response in another study of GIST patients treated with second-line sunitinib.^308^

The prognostic value of DWI-related parameters has been explored in patients with multiple cancer types who received targeted therapies, such as those with unresectable ICC receiving lenvatinib plus PD1 antibody,^307^ recurrent glioblastomas receiving anti-VEGF monotherapy,^309^ colorectal liver metastases receiving bevacizumab,^310^ and NSCLC brain metastases treated with whole-brain radiotherapy and gefitinib.^311^ In general, a higher baseline or post-treatment percentage change of ADC was associated with improved patient outcomes.^300,307,309–315^ These findings indicate that the pretreatment ADC value or post-treatment percentage change of ADC could accurately reflect the therapeutic efficacy of tumor-targeted therapies and predict patient survival. Therefore, the monitoring of ADC could potentially support the optimization of strategies in anticancer treatment.^313^

### Magnetic resonance spectroscopy

The most common metabolites detected by MRS are choline (Cho), lipids, N-acetyl aspartate (NAA), creatine/phosphocreatine (Cr), lactate, and glutamine.^316^ In cancer tissues, the relative concentrations of these compounds are abnormal; for example, in brain tumors, Cho is generally increased, and NAA is decreased compared with the levels in normal brain tissue.^94,317^ Thus, MRS has been commonly used for diagnosing brain tumors and evaluating the therapeutic response. The RTOG 0625/ACRIN 6677 trial, which included patients with recurrent glioblastoma treated with bevacizumab plus chemotherapy, demonstrated that the NAA/Cho level increased and the Cho/Cr level decreased within the enhancing tumor at 2 weeks after treatment compared with the pretreatment levels, indicating potential treatment efficacy.^317^ Similarly, these metabolites demonstrated predictive value in patients with recurrent malignant glioma treated with tamoxifen.^318,319^ In a critical preclinical study, Ros et al. showed that MRS could rapidly assess treatment response to PI3K inhibition in ER^+^ breast cancer mouse models by detecting the changes in lactate and pyruvate levels.^320^ Therefore, this imaging method could help to identify patients who would benefit from current treatments and design new drug combination strategies to counteract treatment resistance.

### Chemical exchange saturation transfer

**Fig. 5:**
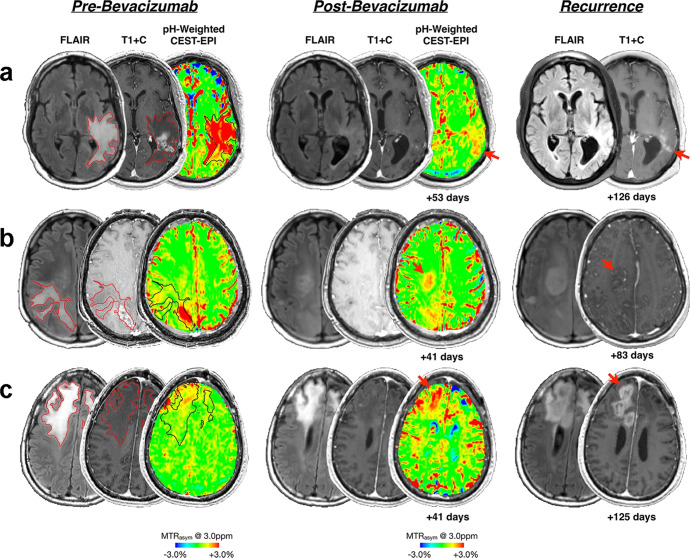
Three patient examples (**a**: patient #4, **b**: patient #8, **c**: patient #11) are demonstrated, with anatomic images (FLAIR and post-contrast T1-weighted images, T1 + C) and pH-weighted CEST-EPI images of MTR_asym_ at 3.0 ppm at three-time points: pre-bevacizumab (baseline), post-bevacizumab (follow-up), and the time of tumor recurrence. Baseline T2 lesion ROIs used for volume and median MTR_asym_ calculation are outlined in red (on FLAIR and post-contrast T1-weighted images) and black (on MTR_asym_ images). The red arrows demonstrate co-localization of residual or newly emerging areas of high acidity (MTR_asym_ at 3 ppm) at the post-treatment time point and the corresponding location of tumor recurrence ~2 months later. CEST-EPI: chemical exchange saturation transfer echoplanar imaging. ROIs: region of interest. FLAIR: fluid-attenuated inversion recovery. (Reproduced from Yao, J. et al. pH-weighted amine chemical exchange saturation transfer echoplanar imaging (CEST-EPI) as a potential early biomarker for bevacizumab failure in recurrent glioblastoma. *J. Neurooncol.* **142**, 587–595 (2019).)

The application of CEST in evaluating treatment response is still in its infancy. In a preclinical study of a human mantle cell lymphoma xenograft model, the acidoCEST technique was used to monitor changes in the tumor extracellular pH (pHe) in response to an mTOR inhibitor, everolimus.^321^ This study showed a significant increase in tumor pHe within 1 day of initiating treatment. Subsequently, acidoCEST MRI identified a decrease in tumor pHe 7 days after initiating treatment.^321^ This contrasts with the findings of an untreated control group from another study performed by the same research group, which showed a consistent decrease in tumor pHe in the same tumor xenograft model.^321^ This reflects the reduction in lactate production due to the inhibition of cellular metabolism by everolimus, as demonstrated in in vitro experiments.^321^ The clinical application of CEST has been explored in small studies of patients with recurrent glioblastoma treated with bevacizumab.^322,323^ Both pH-weighted amine CEST echoplanar imaging^322^ and APT imaging^323^ were potentially useful for predicting the treatment response to bevacizumab and the PFS of patients. Moreover, residual or emerging regions of acidity, as assessed by pH-weighted amine CEST echoplanar imaging, may colocalize to the site of tumor recurrence, which may provide important information for site-specific treatment (see Fig. 5).^322^

### Other techniques

Several other novel techniques have been explored for evaluating the efficacy of treatment in cancer-targeted therapies, such as intravoxel incoherent motion (IVIM) MRI,^324,325^ vascular architecture mapping (VAM) MRI,^326^ arterial spin-labeled (ASL) MRI,^327^ and magnetic resonance elastography (MRE).^328^ In general, these techniques demonstrated the potential to predict the treatment response or long-term outcome of patients. Additionally, MRI biomarkers of the tumor microenvironment, for example, necrosis, hypoxia with/without neovascularization, oxidative phosphorylation, and aerobic glycolysis, are predictive of the treatment response to bevacizumab in patients with glioblastoma.^329^ Gadolinium ethoxybenzyl diethylenetriamine (Gd-EOB-DTPA; a liver-specific contrast agent)-enhanced MRI is an imaging biomarker of OATP1B3, a transporter of Gd-EOB-DTPA.^330^ The expression of OATP1B3 is induced by Wnt/β-catenin mutation. Therefore, EOB-MRI is also considered an imaging biomarker of Wnt/b-catenin mutation/activation, which has been demonstrated to be an important mechanism of resistance to ICIs.^330^ In patients with hepatocellular carcinoma treated with ICIs, those with tumors with hyperintensity in EOB-MRI had significantly shorter PFS than patients with hypointense tumors.^331,332^ Since these studies involved early trials with a small number of patients, the definitive predictive and prognostic value of these new techniques needs to be validated in future studies.

Thus, the MRI parameters, especially their change after treatment, are sensitive to the biological changes induced by cancer-targeted therapies. Together with their radiation-free nature, these MRI techniques are attractive tools in clinical practice for early treatment response assessment and patient prognosis prediction. Their clinical significance in improving patients’ outcomes is worthy of further evaluation in future prospective studies.

### Optical imaging

The evidence on the application of optical imaging for predicting the efficacy of cancer-targeted treatment is from preclinical research but not human studies. BLI has been widely used in the preclinical setting for cancer detection, monitoring disease progression, and assessing the efficacy of anticancer treatment in vivo. The rapid and quantitative assessment of response to cancer treatment by this technique has accelerated drug discovery and development. However, a comprehensive review of the application of this technique in drug development is outside the scope of this paper. Here, we only present some examples to demonstrate how this technique can be used for evaluating the efficacy of cancer-targeted therapy.

**Fig. 6:**
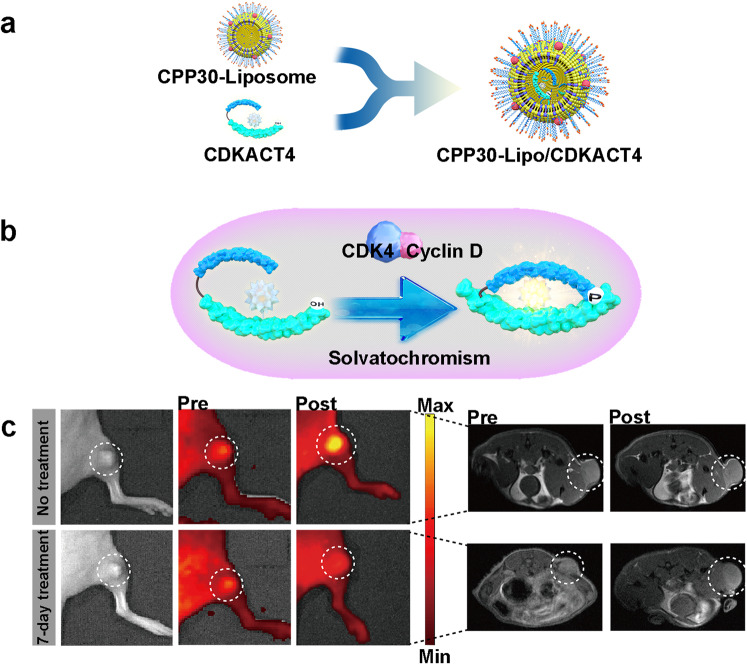
CPP30-Lipo/CDKACT4 reports the CDK4 inhibitor pharmacodynamics in vivo. **a** The chemical structures of molecular probes of CPP30-Lipo/CDKA4. **b** The activation of CPP30-Lipo/CDKA4 within cells. **c** Fluorescence images of mice bearing MCF-7 tumor injected with CPP30-Lipo/CDKACT4 before and after gavage of sterile water for 7 days, or before and after treatment with 150 mg/kg palbociclib daily for 7 days. The fluorescence signals were measured in radiance counts per cm^2^ per second per steradian (p/s/cm^2^/sr) (left). Magnetic resonance imaging (MRI) of mice in (right). (Reproduced from Gao, Y. Y. et al. In vivo visualization of fluorescence reflecting CDK4 activity in a breast cancer mouse model. *MedComm (2020)* **3**, e136 (2022).)

Guo and colleagues developed genetically engineered bioluminescent reporters that reflected the G_1_ phase alternation of the cell cycle.^333^ In vitro and in vivo *experiments, this reporter system was shown* to monitor G_1_ phase arrest caused by a clinically used CDK4/6 inhibitor, palbociclib.^333^ In another preclinical study of a syngeneic murine triple-negative breast cancer model subjected to PD-1 inhibition, BLI could monitor the volume change of luciferase-tagged murine 4T1 tumors after treatment.^334^ Although minimal data from the literature demonstrated the use of FLI for assessing the efficacy of anticancer treatment, several FLI probes have been developed for monitoring the expression of HER2,^335–337^ VEGF/VEGFR,^337,338^ and EGFR.^339–341^ These probes have the potential for the early detection of blocking of these receptors and may be used for early assessment of the efficacy of targeted treatment. Additionally, in a recently published study, Gao and colleagues developed an FLI probe for the real-time monitoring of CDK4 activity. In a hormone receptor-positive/HER2-negative breast cancer xenograft model, they demonstrated that the probe could reflect the therapeutic efficacy of palbociclib before an apparent change in the tumor size (see Fig. 6).^342^ FLI probes have also been constructed for noninvasive, preclinical in vivo evaluation of the efficacy of cancer immunotherapeutics by detecting the presence of either the immune activation-related biomarker granzyme B^343^ or CD8^+^ cytotoxic T lymphocytes.^344^

Interestingly, the clinical application of optical imaging techniques for evaluating the efficacy of cancer-targeted treatment has been explored using a diffuse optical tomography breast imaging system (DOTBIS).^345^ In a proof-of-principle study with seven postmenopausal women with early-stage breast cancer who received pre-surgical treatment with an AKT inhibitor (MK-2206) or an aromatase inhibitor (exemestane or letrozole), DOTBIS was performed on the tumor at baseline and post-therapy. Consistent decreases in DOTBIS-measured total hemoglobin, oxyhemoglobin, deoxyhemoglobin, and water fraction were observed in the tumor after treatment.^345^ This supports the value of further investigation of DOTBIS as a potential tool for assessing the response to cancer-targeted therapies in early-stage breast cancer.

In general, the exploration of optical imaging for treatment efficacy evaluation is in the very early stage. The clinical translation of BLI and FLI is challenging mainly due to their limited tissue penetration and the need for luciferase gene transfection in BLI. On the contrary, the clinical translation of DOTBIS is more feasible as it is a cheap, user-friendly, and repeatable technique.

### Photoacoustic imaging (PAI)

As PAI can use endogenous contrast medium, such as hemoglobin, for real-time and noninvasive imaging, it is well suited for delineating the anatomy of the vasculature and evaluating tumor responses to antiangiogenic therapy.^346^ In a study by Yang et al. ^347^, PAI was used to measure early tumor response in a breast cancer mouse model treated with bevacizumab by clearly identifying the vessels surrounding tumors. By quantitative analysis, PAI parameters including MAP 760, MAP 840, hemoglobin (HbT), and deoxyhemoglobin (HbR) were shown to be significantly reduced 5 days after both high-dose and low-dose bevacizumab treatments compared with the levels in the control group, while no noticeable change in tumor volume was found. In the high-dose group, hypoxia showed negative correlations with these four parameters and CD31 (a marker of the maturation state) showed positive correlations with HbT, HbR, and MAP 760, while VMI (another marker of the maturation state) showed positive correlations with MAP 760 and HbR. Similar conclusions were reached by using photoacoustic tomography (PAT) in breast cancer mouse models treated with bevacizumab^348^ and an ovarian tumor mouse model treated with Trebananib.^349^ Moreover, Pham et al. ^350^ used contrast-enhanced ultrasound (CEUS) and PAI to perform the preclinical evaluation of the efficacy of bevacizumab in combination with CRLX101 (an investigational nanoparticle–drug conjugate). Therefore, PAI has been used as a noninvasive method in preclinical models to measure the effect of antiangiogenic therapy by visualizing vascular regression, normalization, and tumor hypoxia. Its clinical translation in cancer-targeted treatment efficacy evaluation is worthy of exploring, given the fact that PAI has been employed for the early detection of multiple cancers, including breast cancer, melanoma, and prostate cancer.^351^

### Multimodal imaging

Each imaging technique discussed above is associated with certain limitations.^352^ For example, PET/SPECT are associated with poor spatial resolution and risks posed by exposure to radiation, while MRI has relatively low specificity and a long imaging time. In contrast, optical imaging has low spatial resolution and a small penetration depth. Meanwhile, US imaging has poor resolution and subjective results dependent on the particular operator. To overcome these limitations, researchers have attempted to fuse two or more different imaging techniques to create a new imaging mode, also known as multimodal molecular imaging, to obtain more consistent and accurate information.

Currently, most multimodal imaging techniques feature dual modes, involving optical imaging combined with MRI, PET, or SPECT, or PET and SPECT combined with CT or MRI, among others. Multimodal molecular imaging has been utilized in preclinical and clinical research for early diagnosis, disease staging, assessment of therapeutic response, surgical navigation, and prognosis evaluation.

In studies on the application of multimodal molecular imaging in predicting the efficacy of targeted therapy, the focus has mainly been placed on antiangiogenic drugs in preclinical studies. Using a novel metal-based imaging probe, Fe_3_O_4_-DMSA-SMCC-BCZM-^99m^Tc, with the monoclonal antibody bevacizumab radiolabeled with ^99m^Tc for dual-modality SPECT/MR imaging of angiogenesis by targeting VEGF-A was reported.^353^ This approach could be utilized to evaluate the efficacy of potential antiangiogenic drugs. Similar results were found for NIR830-bevacizumab-IONPs with MR and optical imaging.^354^ In a mouse model, Chen et al. detected cancer in vivo using multimodal imaging with photoacoustic and computed tomography (CT), which targeted epidermal growth factor receptors (EGFRs) and ErbB2 and may be used as a new platform for evaluating the response to EGFR- and HER2-targeted therapy.^355^

## Conclusion and future perspectives

This paper reviews studies performed to date on the application of molecular imaging for early prediction of the efficacy of cancer-targeted therapy. Conventional predictive biomarkers for targeted therapy mainly rely on invasive tissue biopsy and subsequent pathological analysis. Since only a limited amount of tissue can be biopsied, as well as the issue of tumor heterogeneity, the conventional histopathological biomarkers obtained from a single lesion are not always predictive of the response to targeted therapy, especially for patients with multiple metastatic tumors. For post-therapy assessment of the efficacy of targeted treatment, the current gold standard is the use of the RECIST criteria, a method based on the change in the tumor size. However, such change often occurs over weeks, or even months, after treatment initiation, which rules out the possibility of detecting treatment resistance soon after it develops.

Against the above background, the novel molecular imaging technique demonstrates more potential than conventional imaging techniques for cancer-targeted therapy in the following aspects: (1) It provides whole-body imaging, such as by immuno-PET,^168^ which better demonstrates the intra-tumoral and inter-tumoral heterogeneity of targeted molecule expression. This enables the prediction of targeted treatment efficacy at the lesion level.^168^ (2) It enables the imaging of early changes (usually several days after treatment) in the functional status of a tumor, which could reflect the response to targeted therapy and enable early detection of treatment resistance and prediction of long-term efficacy. (3) It allows noninvasive monitoring of the changes in the expression of targeted molecules, which is essential for optimizing the treatment strategy during therapy.

To date, almost all studies reporting the application of molecular imaging in cancer-targeted therapy were in early-stage clinical trials with a small number of patients. Although these studies are essential for obtaining preliminary findings on the performance of these advanced imaging techniques for evaluating the efficacy of targeted treatment, their definitive value needs to be confirmed in future studies with larger sample sizes. There is also heterogeneity in the data acquisition among the applied approaches, such as the scanning protocol and timing of scanning, strategy for data analysis, and study endpoints. This makes it difficult to directly compare the data among studies and draw definitive conclusions on the performance of any technique or any parameter for evaluating the efficacy of targeted treatment. There is an urgent need to develop a uniform scanning protocol, data analysis strategy, and study endpoints for each imaging technique, as this is the only way to increase uniformity among studies. This would in turn allow direct data comparison and pooled data analysis and should eventually accelerate the clinical application of molecular imaging for evaluating the efficacy of targeted therapy.

At present, ^18^F-FDG PET-CT, DCE-MRI, and DWI-MRI are the most commonly used techniques for assessing cancer-targeted therapy in clinical practice. These techniques also hold great promise as they enable noninvasive whole-body imaging with deep tissue penetration and high sensitivity for detecting the biological changes after targeted treatment, such as changes in the metabolic activity, perfusion, and diffusion of tumors. Although immune-PET provides target-specific information on tumors and maybe a more helpful tool for evaluating the efficacy of targeted treatment. VEGF, HER2, EGFR, ER, and PD-1/PD-L1 are the most commonly studied targets in this field. Immuno-PET binding to these targets has been applied in early clinical trials to evaluate the anti-cancer targeted treatment efficacy and to predict patient prognosis. Early results demonstrated excellent potential translation of these tools to clinical practice. However, clinical translation of new radiotracers is difficult in many countries, including China, due to regulatory restrictions. Multimodality molecular imaging could be a promising approach to improve the predictive accuracy in evaluating the efficacy of targeted treatment.^300,356–358^ The development of this approach is an essential task in future work.

Along with the increasing use of molecular imaging in cancer management, artificial intelligence (AI) techniques have the potential for several related applications, such as automated interpretation of images and predicting treatment efficacy and patient survival. Information derived from AI may help select the most appropriate treatment for patients and thus improve patient care. An early attempt toward this goal has been reported by Mu and colleagues.^359^ They found that ^18^F-FDG PET-CT images could be used to predict the *EGFR* mutation status using the AI technique of deep learning. A higher EGFR deep learning score (EGFR-DLS) was associated with longer PFS in patients treated with EGFR-TKIs, and a shorter PFS in patients receiving immune checkpoint inhibitors.^359^ The utilization of AI to evaluate the efficacy of targeted treatment and guide clinical decision-making regarding appropriate treatment is expected to increase soon.

Targeted therapy has been one of the cornerstones in treating cancer patients for decades. The rapid development of molecular imaging should improve the early prediction of treatment response to allow early adaptation of patient management and eventually improve patient outcomes. From a perspective point of view, molecular imaging approaches will provide valuable tools to optimize the dosing schedule, determine therapeutic regimes, and monitor the therapeutic response to guide the change in treatment protocol timely if resistance happens. These are important in assisting the clinical decision-making of multidisciplinary treatment, including targeted therapy.

## Supplementary information

copyright of Figure 5
